# Novel analogues of a nonnucleoside SARS-CoV-2 RdRp inhibitor as potential antivirotics

**DOI:** 10.3762/bjoc.20.91

**Published:** 2024-05-06

**Authors:** Luca Julianna Tóth, Kateřina Krejčová, Milan Dejmek, Eva Žilecká, Blanka Klepetářová, Lenka Poštová Slavětínská, Evžen Bouřa, Radim Nencka

**Affiliations:** 1 Department of Organic Chemistry, Faculty of Science, Charles University, Hlavova 2030/8, 128 43 Prague, Czech Republic; 2 Institute of Organic Chemistry and Biochemistry of the Czech Academy of Sciences, Flemingovo náměstí 542/2, 166 10 Prague, Czech Republic

**Keywords:** antivirotics, nonnucleotide inhibitor, RNA-dependent RNA polymerase, SARS-CoV-2

## Abstract

The RNA-dependent RNA polymerase (RdRp) represents a prominent target in the discovery and development of new antivirotics against RNA viruses, inhibiting the replication process. One of the most targeted RNA viruses of the last years is, without doubt, SARS-CoV-2, the cause of the recent COVID-19 pandemic. HeE1-2Tyr, a known inhibitor of flaviviral RdRp, has been discovered to also have antiviral potency against this coronavirus. In this study, we report three distinct modifications of HeE1-2Tyr: conversion of the core from a benzothiazole to a benzoxazole moiety and two different scaffold simplifications, respectively. We provide a novel synthetic approach and, in addition, evaluate the final molecules in an in vitro polymerase assay for biological activity.

## Introduction

Epidemics caused by various viral infections, such as AIDS, Zika fever, Dengue fever, or Ebola, are a constant threat to communities of all sizes [1]. The COVID-19 pandemic, caused by the newly emerged severe acute respiratory syndrome coronavirus type 2 (SARS-CoV-2), has put an enormous pressure on the healthcare system worldwide and called for immediate action in prevention and treatment, which in turn required the discovery of new effective therapeutic options. It seems to be clear that the widespread use of vaccines is able to stop the acute phase of the pandemic. However, antiviral therapy for COVID-19 is indispensable in case of vaccine failure, virus mutation or suppressed immunity of some patients [2].

SARS-CoV-2 is part of the *Coronaviridae* family, a group of enveloped +ssRNA viruses. The genome can directly act as a viral messenger RNA and encodes essential enzymes for replication [3]. Inhibiting these nonstructural proteins that are part of the replication complex has already shown great success in antiviral therapy [4–7].

The viral RNA-dependent RNA polymerase (RdRp) is encoded in all RNA viruses and plays a crucial role in viral RNA replication. In the proteome of SARS-CoV-2, the catalytic subunit nsp12, expressed together with the cofactors nsp7 and nsp8, constitutes the RdRp [8]. RdRp is usually targeted by nucleotide analogue inhibitors (NAIs) [9]. This class of antivirals can inhibit the replication by acting as a delayed chain terminator or by causing genetic corruption in the viral RNA and includes the first FDA-approved antiviral drugs in the therapy of COVID-19 patients, remdesivir [10] and molnupiravir [11]. The usability of NAIs may largely depend on the metabolic activation, and they also compete with the intracellular pool of natural nucleoside triphosphates (NTPs). Nonnucleotide analogue inhibitors (NNAIs) do not face these challenges as they bind to both active but also allosteric sites of the RdRp, and therefore they represent a promising NAI alternative [12].

Since the beginning of the pandemic, a variety of heterocyclic small molecules – either of natural or synthetic origin – was reported as promising inhibitors of the SARS-CoV-2 RdRp [13–15]. However, compounds with a sufficient combination of high potency and suitable pharmacokinetic properties are still scarce. Recently, many studies have been focusing on drug repurposing or screening libraries of already approved biologically active compounds [16–17]. This approach might represent a very promising strategy in the case of targeting the coronaviral RdRp due to the highly conserved structure of the polymerase, not only across the CoV group but also in other RNA viruses [13]. A great example of this phenomenon is remdesivir, which was originally developed as a therapeutic agent against Ebola virus [18–19].

HeE1-2Tyr (**1**) was originally identified by Tarantino et al. [20] as a potent inhibitor of RdRp from all members of the genus *Orthoflavivirus* [20–23] and was crystallized in complex with the RdRp from DENV-3 [20]. In 2021, our group reported this compound to also exhibit inhibitory activity against feline infectious peritonitis virus (FIPV) and SARS-CoV-2 RdRp and to hinder viral replication in cell-based antiviral assays [24]. That study highlighted the beneficial role of the tyrosine residue and the indispensable role of the C-2 substitution.

**Figure 1:**
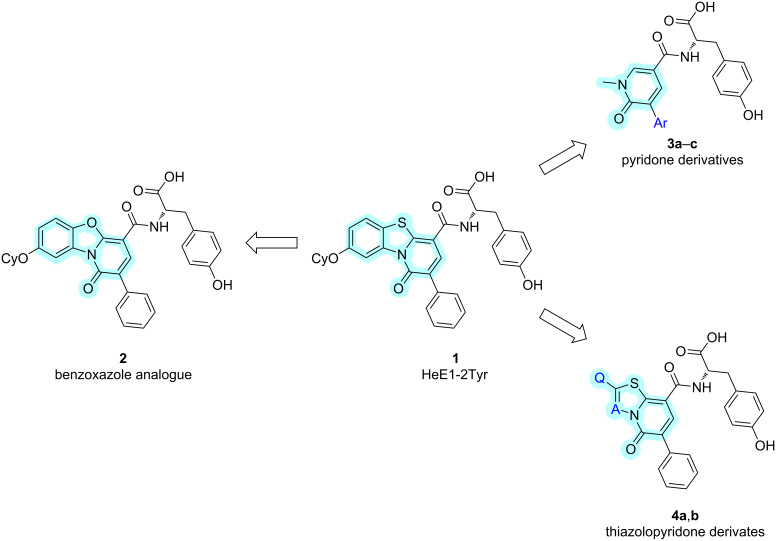
Structure of HeE1-2Tyr (**1**) and of the derivatives synthesized in this work.

In this work, we report the synthesis and biological evaluation of further analogues of HeE1-2Tyr (**1**) against the SARS-CoV-2 RdRp. We focused on the modification of the central heterocyclic core and on the simplification and truncation of the relatively large molecule **1** (Figure 1).

In this work, replacing the sulfur atom with a (bio)isosteric oxygen atom yielded two novel structural analogues, whilst our effort towards more simple molecules led to a series of pyridone derivatives. Out of these, **3a** had already been synthesized by a different approach [22]. However, this study presents a novel and notably simpler synthetic route. Furthermore, as part of the systematic truncation of the core, we synthesized thiazolopyridone and thiadiazolopyridone derivatives because molecules based on these cores have already shown promising antimicrobial activities [25–26].

## Results and Discussion

### Synthesis of HeE1-2Tyr (**1**) structural analogues

#### Modification of the core: synthesis of pyridobenzoxazole derivatives

**Scheme 1:**
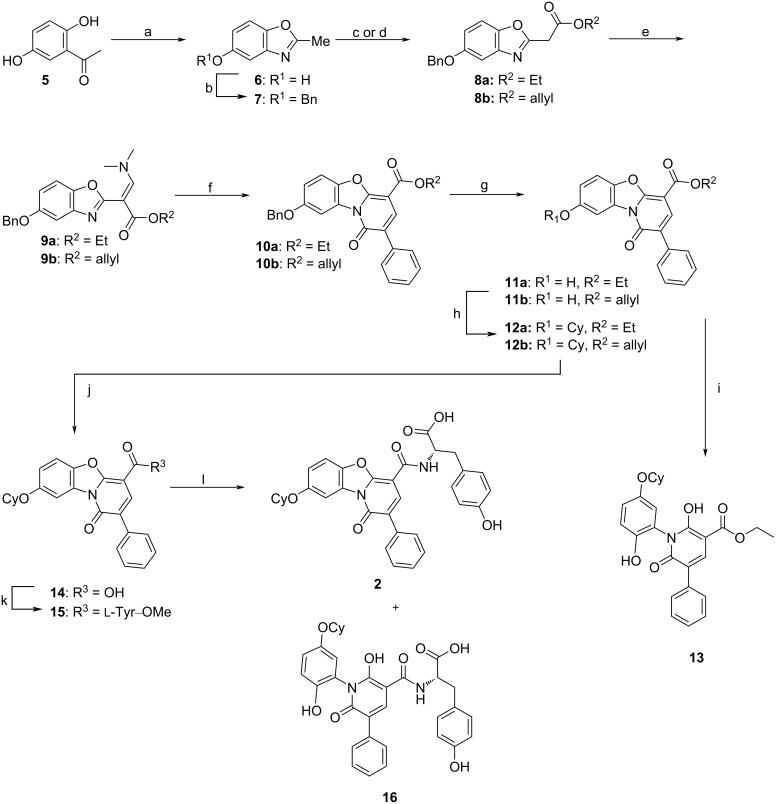
Synthetic pathway to benzoxazole analogue **2** of HeE1-2Tyr (**1**). Reagents and conditions: a) TMSN_3_, TfOH, DCM, rt, overnight, b) BnBr, NaH, DMF, 0 °C to rt, 1 h, c) (R = Et) diethyl carbonate, LiHMDS, THF, −78 to 0 °C, 1 h, d) (R = allyl) allyl chloroformate, LiHMDS, THF, −78 to 0 °C, 1 h, e) POCl_3_, DMF, 90 °C, 30 min, f) (BnCO)_2_O, 100 °C, 1.5 h, g) methanesulfonic acid, DCM, 0 °C to rt, 4 h, h) CyOH, PPh_3_, DIAD, 1,4-dioxane, 50 °C, overnight, i) (R = Et) NaOH, H_2_O, EtOH, 75 °C, 3 h, j) (R = allyl) Et_3_SiH, PPh_3_, Pd(OAc)_2_, ACN, rt, k) H–ʟ-Tyr–OMe, HOBt, EDCI, TEA, DCM, DMF, rt, 12 h, l) LiOH⋅H_2_O, H_2_O, 1,4-dioxane, rt, 45 min. Cy = cyclohexyl.

The synthesis of the pyridobenzoxazole derivative **2** was designed based on the modified approach published by Dejmek et al. (Scheme 1) [24].

In this work, we first synthesized the intermediate **6** from readily available 2′,5′-dihydroxyacetophenone (**5**) following a published procedure [27]. This compound was then easily converted to the suitably decorated benzoxazole derivative **12a**. The benzoxazole core showed increased sensitivity towards a basic environment, resulting in the ring-opened side product **13** through saponification of the ester function of compound **12a**. The identification of this side product proved to be challenging due to insufficient evidence provided even by meticulous NMR analysis and eventually had to be confirmed by X-ray crystallography (Figure S1, Supporting Information File 1). Changing the ester function from an ethyl to an allyl group enabled a very mild cleavage using a Pd-mediated reaction with triethylsilane [28], and thus avoiding the use of base, leading to the desired intermediate **14** in good yield. Compound **14** was then coupled with ʟ-tyrosine methyl ester followed by deprotection of the amino acid carboxyl group by LiOH⋅H_2_O. As in the previous base-mediated saponification, here we also received a product of the benzoxazole ring-opening reaction, namely **16**.

#### Simplification of the hit molecule: synthesis of pyridone derivatives

**Scheme 2:**
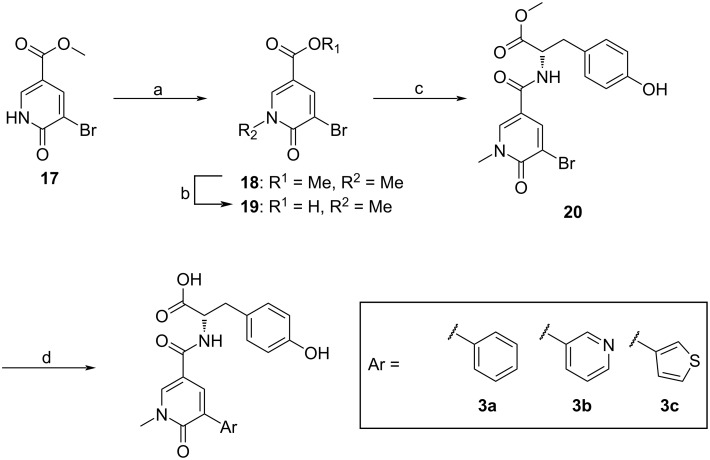
Synthetic pathway to pyridone derivatives **3a**–**c** of HeE1-2Tyr (**1**). Reagents and conditions: a) MeI, K_2_CO_3_, DMF, rt, 2.5 h, b) LiOH⋅H_2_O, H_2_O, 1,4-dioxane, rt, 15 min, c) H–ʟ-Tyr–OMe, HOBt, EDCI, TEA, DCM, DMF, rt, 12 h, d) ArB(OH)_2_, Pd(dppf)Cl_2⋅_CH_2_Cl_2_, Cs_2_CO_3_, DMF, H_2_O, 80 °C, overnight.

We decided to simplify the relatively large structure of HeE1-2Tyr (**1**) in order to obtain smaller, more accessible inhibitors with similar or better properties. The employed novel synthetic strategy leading to pyridones bearing different aryl substituents is described in Scheme 2. During the Suzuki–Miyaura cross-coupling reaction, which introduced the substituents in the C-5 position, the methyl ester protection of the amino acid moiety was also cleaved, leading directly to the final compounds **3a** (first reported by Cannalire et al. [22]) and **3b**,**c**.

#### Simplification of the hit molecule: synthesis of thiazolopyridone derivatives

**Scheme 3:**
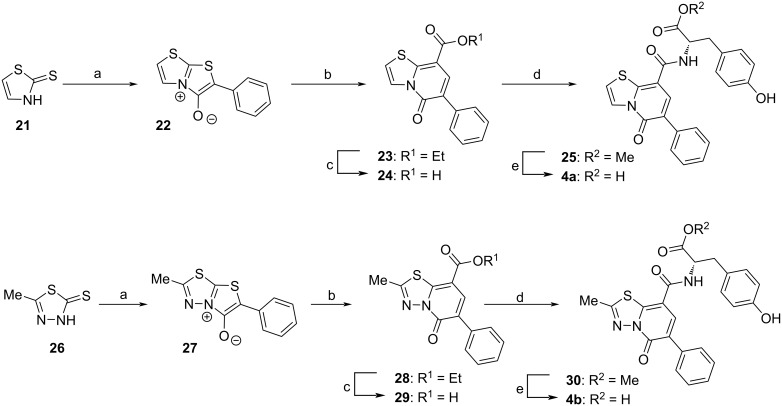
Synthetic pathway to thiazolopyridone derivatives **4a**,**b** of HeE1-2Tyr (**1**). Reagents and conditions: a) 2-bromo-2-phenylacetyl chloride, TEA, THF, rt, 1 h, b) ethyl acrylate, toluene, 110 °C, 24 h, c) NaOH, H_2_O, MeOH, 70 °C, 2 h, d) H–ʟ-Tyr–OMe, HOBt, EDCI, TEA, DCM, DMF, rt, 12 h, e) LiOH⋅H_2_O, H_2_O, 1,4-dioxane, rt, 2 h.

Further, a novel type of inhibitor containing a thiazolopyridone core and the corresponding azo derivative, namely **4a**,**b**, were synthesized. The synthetic route was designed based on the work reported by Potts et al. [29] and is described in Scheme 3. The 2-bromo-2-phenylacetyl chloride, necessary for the first step of the synthesis, was prepared from readily available phenylacetic acid [30–31]. The reaction with the 5-membered heterocycles **21** and **26**, respectively, led to two crucial mesoionic compounds, **22** and **27**. The recrystallized intermediates then underwent a formal cycloaddition with ethyl acrylate, followed by the elimination of H_2_S, forming the desired heterocyclic core structures (intermediates **23** and **28**, respectively). A subsequent saponification step led to the corresponding carboxylic acids **24** and **29**, and from there, the desired final compounds **4a**,**b** were obtained in a straightforward two-step synthesis.

### Biochemical study: inhibition of SARS-CoV-2 RdRp

**Figure 2:**
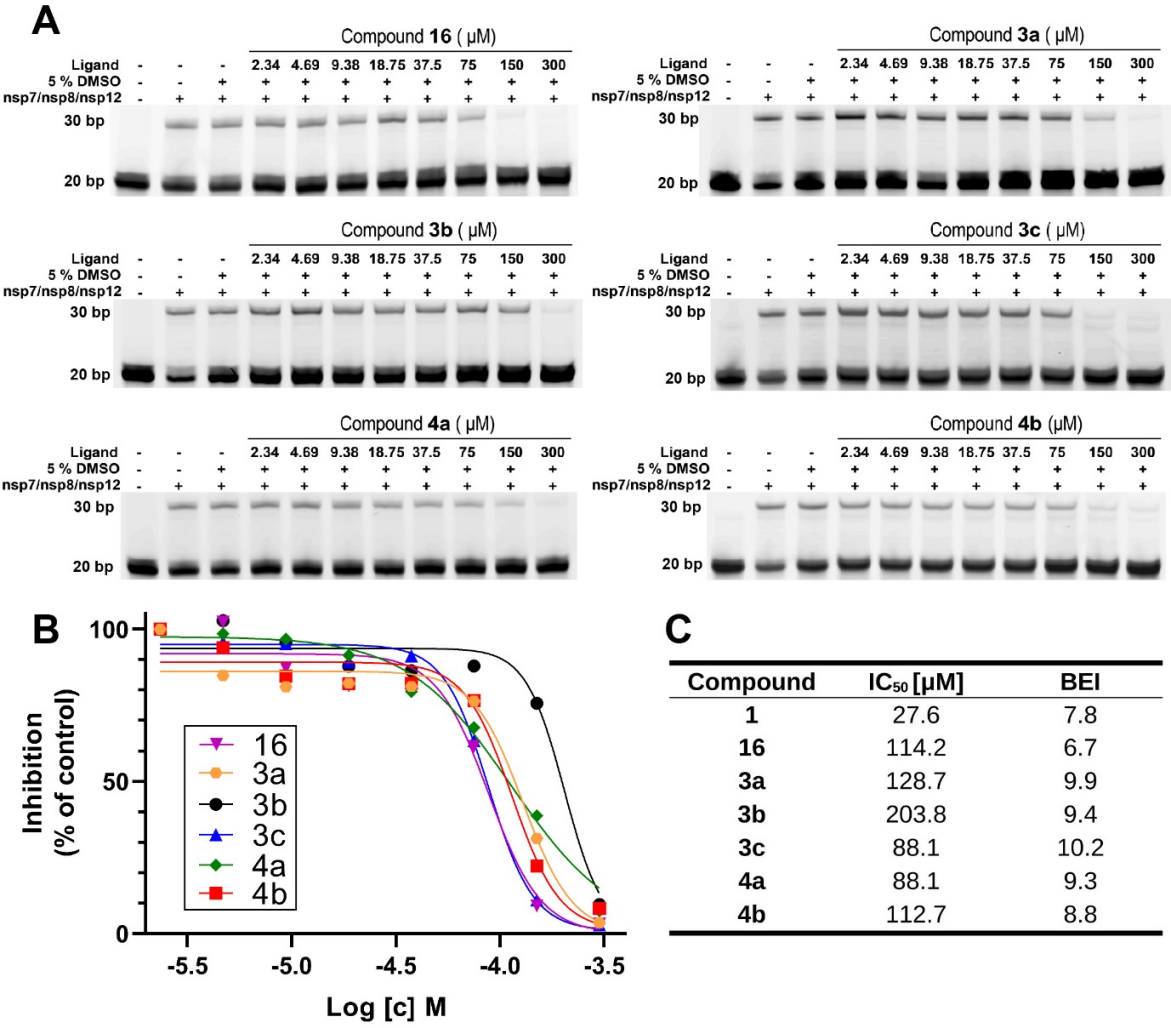
Analysis of inhibitory activity against SARS-CoV-2 RdRp using primer extension assay. A) Gel-based polymerase assay with a constant concentration of fluorescently labeled template/primer RNA (0.5 µM) and the polymerase complex (nsp7, nsp8 3 µM and nsp12 1 µM), along with increasing concentrations of compounds as indicated at the top. Reactions were initiated by adding 10 µM NTPs and run for 1 h at 30 °C. The reactions were stopped by adding stop buffer, and the products were separated on a 20% denaturing gel. B) Graphical representation of the inhibitory activity of selected compounds evaluated from the gels obtained in the primer extension assay. The percentage of inhibition (against control) was plotted against the logarithm of the concentration of compounds. The results were fitted to sigmoidal dose–response curves. C) The IC_50_ values were determined using the GraphPad algorithm (IC_50_ value of compound **1** was published by Dejmek et al. [24]), the BEI was calculated using the function pIC_50_ [mol/L]/MW [kDa].

We aimed to determine the inhibitory activity of the final compounds **2**, **16**, **3a**–**c** and **4a**,**b** against SARS-CoV-2 RdRp. The RdRp was prepared recombinantly, and the inhibitory activity was measured using a primer extension polymerase assay. This assay was also used to determine the IC_50_ values (Figure 2 and Figure S2, Supporting Information File 1). The benzoxazole analogue **2** was devoid of any activity, while the ring-opened derivative **16** showed inhibition with IC_50_ = 114.2 μM. It seemed that the significantly smaller size of the oxygen atom in the benzoxazole derivative compared to sulfur in HeE1-2Tyr (**1**) led to an unfavorable molecular shape, while the analogue with the open ring was able to compensate this difference. The pyridone derivatives **3a**–**c** exerted an activity resulting in IC_50_ values of 128.7 μM, 203.8 μM and 88.1 μM, respectively, highlighting that even significantly truncated molecules are capable of RdRp inhibition. A thiophene substituent in position 5 (i.e., **3c**) proved to be the most successful modification. The potential of the truncated derivatives was further illustrated by the thiazolopyridone and thiadiazolopyridone compounds **4a**,**b**, which showed inhibition with IC_50_ = 88.1 μM and 128.7 μM, respectively. Even though the measured inhibitory concentration was higher than that of HeE1-2Tyr (**1**), it must be considered that the synthesized ligands were significantly smaller in size. Normalization of the obtained results using the binding efficiency index (BEI) [32] suggest that both ligand types, **3a**–**c** and **4a**,**b**, bind more efficiently to the SARS-CoV-2 RdRp when compared to **1**.

## Conclusion

In this study, novel analogues of the antiviral HeE1-2Tyr (**1**) were synthesized and evaluated with respect to the in vitro inhibitory activity towards SARS-CoV-2 RdRp. To obtain the benzoxazole analogue, a new synthetic strategy avoiding base-mediated hydrolysis was successfully applied. For the simplified structural derivatives, the applied routes were optimized for maximal efficacy of the synthetic work. Regarding the inhibitory activity, six of the novel compounds showed inhibition in the fluorescence-based primer extension assay. The two simplified molecules were the most promising inhibitors, with an IC_50_ value below 90 µM, and the compounds **3a**–**c** and **4a**,**b** exerted stronger BEIs than **1**. The obtained results provide important information about the structural requirements for the heterocyclic inhibitors based on HeE1-2Tyr (**1**), which we will use in the design of further generations of such antivirals.

## Supporting Information

File 1 Experimental procedures, spectra and X-ray data.

## Data Availability

The data that supports the findings of this study is available from the corresponding author upon reasonable request.
