# Supplementary material for: Novel analogues of a nonnucleoside SARS-CoV-2 RdRp inhibitor as potential antivirotics
Source: Beilstein J Org Chem. 2024 May 6;20:1029–36. doi: 10.3762/bjoc.20.91 (PMC11092066; doi:10.3762/bjoc.20.91)
Supplement: File 1 — Experimental procedures, spectra and X-ray data. [file Beilstein_J_Org_Chem-20-1029-s001.pdf]

BEILSTEIN JOURNAL OF ORGANIC CHEMISTRY

Supporting Information

for

Novel analogues of a nonnucleoside SARS-CoV-2 RdRp inhibitor as potential antivirotics

Luca Julianna Tóth, Kateřina Krejčová, Milan Dejmek, Eva Žilecká, Blanka Klepetářová, Lenka Poštová Slavětínská, Evžen Bouřa and Radim Nencka

Beilstein J. Org. Chem. **2024,** *20,* 1029–1036. doi:10.3762/bjoc.20.91

Experimental procedures, spectra and X-ray data

License and Terms: This is a supporting information file under the terms of the Creative Commons Attribution License (https://creativecommons.org/licenses/by/4.0). Please note that the reuse, redistribution and reproduction in particular requires that the author(s) and source are credited and that individual graphics may be subject to special legal provisions.
The license is subject to the *Beilstein Journal of Organic Chemistry* terms and conditions: (https://www.beilstein-journals.org/bjoc/terms)

Table of contents

- **Experimental section: chemistry** S2
    - Methods and instrumentation S2
    - Synthesis of inhibitors S2
- **^{1}H NMR, ^{13}C NMR, HRMS spectra and UPLC–MS traces of the final compounds** S15
- **X-ray crystallography: crystal data and structure** S29
    - Crystal data and crystal structure of compound **13** S29
- **Biochemical study: inhibition of SARS-CoV-2 RdRp** S30
    - Protein expression and purification S30
    - Primer extension polymerase assay S30
    - Fluorescence-based primer extension assay of compound **2** S30
- **References** S31

Experimental section: chemistry

Methods and instrumentation

Starting compounds and reagents were purchased from commercial suppliers (Sigma-Aldrich, Fluorochem, Acros Organics, TCI) and used without further purification. Analytical TLC was performed on silica-gel-precoated aluminum plates with a fluorescent indicator (Merck 60 F_{254}). Mass spectra, UV absorption, and purity of compounds were measured on a Waters UPLC–MS system, consisting of the Waters UPLC H-Class Core System (Waters Acquity UPLC BEH C18 1.7 mm column, 2.1 × 100 mm), the Waters Acquity UPLC PDA detector and the Waters SQD2 mass spectrometer. The universal LC method was used (eluent H_2O/CH_3CN, gradient 0–100%, run length 7 min) in conjunction with the MS method (ESI+ and/or ESI−, cone voltage 30 V, mass detector range 100–1000 Da). NMR spectra were recorded on a Bruker Avance III HD (^{1}H at 400 MHz) spectrometer using DMSO-d_6, $CDCl_3$ or CD_3OD as a solvent, and the solvent signal as a reference. Chemical shifts (δ) and coupling constants (*J*) were expressed in ppm and Hz, respectively. All structures were confirmed using combination of 1D and 2D NMR (^{1}H,^{13}C-APT, ^{1}H,^{1}H-COSY, ^{1}H,^{13}C-HSQC, ^{1}H,^{13}C-HMBC) techniques. Standard pulse programs from the library of the spectrometer were used, and gradient selection was used in the 2D experiments. The HRMS spectra were performed with an LTQ Orbitrap XL instrument (Thermo Fisher Scientific) using ESI. The structure of compound **13** was confirmed by X-ray crystallography (Figure S1). Flash column chromatography was performed on silica gel or C18 reversed phase silica gel, using Teledyne ISCO CombiFlash Nextgen.

Synthesis of inhibitors

2-Methylbenzo[*d*]oxazol-5-ol (**6**) [1]

To a solution of 2′,5′-dihydroxyacetophenone (**5**, 10.0 g, 65.73 mmol, 1.0 equiv) in dry DCM (200.0 mL) were added $TMSN_3$ (13.0 mL, 98.59 mmol, 1.5 equiv) and TfOH (8.7 mL, 98.59 mmol, 1.5 equiv) at room temperature, and the mixture was stirred overnight. The reaction mixture was diluted with water and the resulting solution was extracted with EtOAc. The combined organic layers were washed with brine, dried over anhydrous Na_2SO_4, filtered, and concentrated under reduced pressure to obtain the pale-red solid product **6** (9.44 g, 96%). NMR spectra were consistent with literature.

^{1}H NMR (401 MHz, $CDCl_3$) *δ* 7.30 (d, *J* = 8.7 Hz, 1H), 7.18 (d, *J* = 2.4 Hz, 1H), 6.86 (dd, *J* = 8.8, 2.5 Hz, 1H), 5.69 (bs, 1H), 2.63 (s, 3H).
^{13}C NMR (101 MHz, $CDCl_3$) *δ* 165.30, 153.92, 145.41, 141.17, 113.64, 110.73, 104.99, 14.62.

ESI MS *m/z* (%): 150 (100) [M+H$^+$]

5-(Benzyloxy)-2-methylbenzo[*d*]oxazole (**7**)

To a solution of **6** (8.90 g, 59.67 mmol, 1.0 equiv) in dry DMF (143.0 mL), NaH (60% in mineral oil, 2.69 g, 67.20 mmol, 1.1 equiv) was added in several portions under Ar atmosphere at 0 °C. After 15 min, BnBr (8.4 mL, 70.38 mmol, 1.2 equiv) was added, then the suspension was allowed to warm to ambient temperature and stirred for 1 h, when TLC analysis (cyclohexane/EtOAc/MeOH 10:8:2) showed no remaining starting material. The reaction mixture was quenched with NH_4Cl, diluted with water and extracted with EtOAc. The organic phase was washed with brine, dried over anhydrous Na_2SO_4 and evaporated to give a brown solid, which was purified by reversed phase flash column chromatography (ACN in water, from 10% to 100%) to yield the product **7** as an off-white powder (14.28 g, quantitative yield).

^{1}H NMR (401 MHz, $CDCl_3$) δ 7.47 – 7.43 (m, 2H), 7.39 (ddd, *J* = 7.9, 6.9, 1.0 Hz, 2H), 7.35 – 7.30 (m, 1H, and d, *J* = 8.9 Hz, 1H), 7.21 (d, *J* = 2.5 Hz, 1H), 6.96 (dd, *J* = 8.9, 2.5 Hz, 1H), 5.10 (s, 2H), 2.61 (s, 3H).
^{13}C NMR (101 MHz, $CDCl_3$) δ 164.81, 156.25, 145.90, 142.41, 137.01, 128.73, 128.14, 127.67, 113.67, 110.45, 104.31, 71.03, 14.74.

ESI MS *m/z* (%): 240 (100) [M+H$^+$]

Ethyl 2-(5-(benzyloxy)benzo[*d*]oxazol-2-yl)acetate (**8a**)

To a solution of **7** (3.72 g, 15.55 mmol, 1.0 equiv) in dry THF (65.0 mL), LiHMDS (1 M solution in THF, 34.2 mL, 34.20 mmol, 2.2 equiv) was added at −78 °C under Ar atmosphere and stirred for 30 min. Diethyl carbonate (2.3 mL, 18.66 mmol, 1.2 equiv) was added at −78 °C, and the solution was stirred for 40 min at 0 °C. The reaction was treated with saturated NH_4Cl solution, and the reaction mixture was diluted with EtOAc and water. The aqueous phase was extracted with EtOAc, and the organic phase was washed with brine, dried with anhydrous Na_2SO_4, and the solvent was evaporated under reduced pressure. The residue was purified by reversed phase flash column chromatography (ACN in water, from 10% to 100%) to yield the product **8a** as a brown oil that solidified upon standing overnight (2.50 g, 52%).

^{1}H NMR (401 MHz, $CDCl_3$) δ 7.47 – 7.36 (m, 4H, and d, 1H, *J* = 8.9 Hz), 7.35 – 7.30 (m, 1H), 7.26 (d, *J* = 2.6 Hz 1H), 7.02 (dd, *J* = 8.9, 2.6 Hz, 1H), 5.10 (s, 2H), 4.24 (q, *J* = 7.1 Hz, 2H), 3.98 (s, 2H), 1.28 (t, *J* = 7.2 Hz, 3H).
^{13}C NMR (101 MHz, $CDCl_3$) δ 167.14, 160.48, 156.46, 146.10, 142.08, 136.92, 128.75, 128.18, 127.66, 114.68, 110.94, 104.64, 71.03, 62.05, 35.53, 14.23.

ESI MS *m/z* (%): 312 (100) [M+H$^+$]

Ethyl (*E*)-2-(5-(benzyloxy)benzo[*d*]oxazol-2-yl)-3-(dimethylamino)acrylate (**9a**)

$POCl_3$ (1.5 mL, 16.06 mmol, 2.0 equiv) was added carefully to ice-cooled DMF (1.9 mL, 24.09 mmol, 3.0 equiv), and the resulting mixture was stirred for 30 min at ambient temperature. To the formed Vilsmeier reagent, a solution of **8a** (2.50 g, 8.03 mmol, 1.0 equiv) in DMF (10.0 mL) was added dropwise, and the reaction mixture was heated at 90 °C under stirring for 30 min. After cooling, the mixture was treated with ice water, basified to pH 8 with solid K_2CO_3, and extracted several times with EtOAc. The combined organic layers were washed with brine, dried with anhydrous Na_2SO_4, and the solution was evaporated to dryness giving the reactive intermediate **9a** as a dark orange oil (3.22 g, quantitative yield), which was used in the next reaction without further purification.

ESI MS *m/z* (%): 367 (100) [M+H$^+$]

Ethyl 8-(benzyloxy)-1-oxo-2-phenyl-1*H*-benzo[4,5]oxazolo[3,2-*a*]pyridine-4-carboxylate (**10a**)

A mixture of **9a** (3.12 g, 8.52 mmol, 1.0 equiv) and phenylacetic anhydride (4.78 g, 18.80 mmol, 2.2 equiv) was heated at 100 °C under stirring for 1.5 h. After cooling, the reaction mixture was dissolved in $CHCl_3$, and MeOH was added, giving the product **10a** as a light-yellow precipitate (1.89 g, 51%).

^{1}H NMR (400 MHz, $CDCl_3$) δ 8.40 (d, J = 2.7 Hz, 1H), 8.32 (s, 1H), 7.78 – 7.72 (m, 2H), 7.54 (d, J = 9.0 Hz, 1H), 7.49 – 7.44 (m, 4H), 7.43 – 7.31 (m, 4H), 7.15 (dd, J = 9.1, 2.6 Hz, 1H), 5.16 (s, 2H), 4.45 (q, J = 7.1 Hz, 2H), 1.44 (t, J = 7.1 Hz, 3H).
^{13}C NMR (101 MHz, $CDCl_3$) δ 163.12, 158.87, 156.74, 154.83, 141.68, 139.01, 136.29, 135.62, 128.80, 128.75, 128.54, 128.37, 127.90, 127.86, 127.74, 122.53, 116.04, 111.68, 102.71, 92.18, 71.14, 61.24, 14.62.

ESI MS *m/z* (%): 440 (100) [M+H$^+$]

Ethyl 8-hydroxy-1-oxo-2-phenyl-1*H*-benzo[4,5]oxazolo[3,2-*a*]pyridine-4-carboxylate (**11a**)

To a solution of **10a** (1.88 g, 4.28 mmol, 1.0 equiv) in DCM (50.0 mL) was added methanesulfonic acid (5.6 mL, 85.56 mmol, 20.0 equiv), and the reaction mixture was stirred at 0 °C for 2 h. Monitoring by TLC (cyclohexane/EtOAc, 1:1) showed significant amount of starting material, thus the reaction mixture was warmed to ambient temperature and stirred for another 2 h. After the completion of the reaction, the volatiles were evaporated, the residue was dissolved in EtOAc and washed with saturated solution of $NaHCO_3$ three times. The aqueous phase was washed with EtOAc, the combined organic phase was washed with brine, dried over anhydrous Na_2SO_4 and evaporated. The product with free hydroxy group (i.e., **11a**) was obtained as a light-yellow solid (1.44 g, 97%), and was used in the next reaction without further purification.

ESI MS *m/z* (%): 350 (100) [M+H$^+$]

Ethyl 8-(cyclohexyloxy)-1-oxo-2-phenyl-1*H*-benzo[4,5]oxazolo[3,2-*a*]pyridine-4-carboxylate (**12a**)

To a solution of **11a** (800 mg, 2.29 mmol, 1.0 equiv) in dry dioxane (25.0 mL), cyclohexanol (478 µL, 4.62 mmol, 2.0 equiv), PPh_3 (1.29 g, 4.91 mmol, 2.1 equiv) and DIAD (884 µL, 4.51 mmol, 2.0 equiv) were sequentially added at 0 °C, and the reaction mixture was stirred at 50 °C overnight. Volatiles were evaporated, and the residue was purified by normal phase flash column chromatography (EtOAc with 10% MeOH in cyclohexane, from 0% to 40%). The product **12a** (988 mg, quantitative yield) was obtained as a yellow oil, which solidified upon standing overnight.

^{1}H NMR (401 MHz, $CDCl_3$) δ 8.30 (s, 1H), 8.28 (d, J = 2.6 Hz, 1H), 7.76 – 7.70 (m, 2H), 7.52 (d, J = 9.0 Hz, 1H), 7.49 – 7.43 (m, 2H), 7.39 – 7.34 (m, 1H), 7.07 (dd, J = 9.0, 2.6 Hz, 1H), 4.45 (q, J = 7.1 Hz, 2H), 4.39 – 4.32 (m, 1H), 2.04 – 1.96 (m, 2H), 1.84 – 1.76 (m, 2H), 1.62 – 1.49 (m, 4H), 1.43 (t, 3H, J = 7.1 Hz), 1.40 – 1.29 (m, 2H).
^{13}C NMR (101 MHz, $CDCl_3$) δ 163.15, 158.93, 155.86, 154.86, 141.38, 138.99, 135.68, 128.77, 128.54, 127.86, 127.83, 122.50, 117.25, 111.60, 103.98, 92.13, 76.88, 61.21, 31.73, 25.66, 23.73, 14.61.

ESI MS *m/z* (%): 432 (100) [M+H$^+$]

Ethyl 1-(5-(cyclohexyloxy)-2-hydroxyphenyl)-2-hydroxy-6-oxo-5-phenyl-1,6-dihydropyridine-3-carboxylate (**13**)

To a solution of **12a** (500 mg, 1.16 mmol, 1.0 equiv) in EtOH (42.0 mL), 10% aqueous NaOH solution was added (10.5 mL), and the reaction mixture was heated at 75 °C for 3 h. The reaction mixture was cooled to room temperature, evaporated to half of the volume, and poured on ice water. The pH was set to 3–4 via addition of HCl (1 M), and the product was extracted with EtOAc. The organic phase was washed with brine, dried with anhydrous Na_2SO_4 and evaporated. The brown sticky residue was purified by reversed phase flash column chromatography (ACN in H_2O, from 10% to 100%) to obtain the product **13** as a dark purple solid (102 mg, 22%).

^{1}H NMR (401 MHz, DMSO-d_6) δ 9.39 (bs, 1H), 7.90 (s, 1H), 7.64 – 7.56 (m, 2H), 7.38 (dd, J = 8.4, 6.7 Hz, 2H), 7.34 – 7.27 (m, 1H), 6.92 – 6.83 (m, 3H), 4.37 (q, J = 7.1 Hz, 2H), 4.15 (tt, J = 8.8, 3.7 Hz, 1H), 1.90 (dt, J = 12.1, 3.8 Hz, 2H), 1.74 – 1.61 (m, 2H), 1.56 – 1.45 (m, 1H), 1.45 – 1.14 (m, 5H), 1.34 (t, J = 7.1 Hz, 3H).
^{13}C NMR (101 MHz, DMSO-d_6) δ 169.31, 162.50, 160.29, 149.96, 146.60, 136.26, 136.06, 128.10, 128.05, 127.08, 122.25, 120.37, 118.1[a], 116.85, 116.81, 88.8[a], 75.17, 61.57, 31.39, 25.17, 23.20, 14.17.

ESI MS *m/z* (%): 450 (100) [M+H$^+$]

[a]The compound was measured as tautomeric mixture.

Allyl 2-(5-(benzyloxy)benzo[*d*]oxazol-2-yl)acetate (**8b**)

To a solution of **7** (5.00 g, 21.00 mmol, 1.0 equiv) in dry THF (100.0 mL), LiHMDS (1 M solution in THF, 46.0 mL) was added at −78 °C under Ar atmosphere and stirred for 30 min. Allyl chloroformate (2.7 mL, 25.08 mmol, 1.2 equiv) was added, and the solution was stirred for 45 min at 0 °C. The reaction was treated with saturated NH_4Cl solution, the reaction mixture was diluted with EtOAc and water, the aqueous phase was further extracted with EtOAc. The combined organic phases were washed with brine, dried over anhydrous Na_2SO_4, filtered, and the solvent was evaporated under reduced pressure to obtain the product **8b** as a brown solid (6.76 g, quantitative yield).

^{1}H NMR (401 MHz, DMSO-d_6) δ 7.62 (d, *J* = 8.9 Hz, 1H), 7.50 – 7.45 (m, 2H), 7.43 – 7.37 (m, 2H), 7.37 (d, *J* = 2.6 Hz, 1H), 7.35 – 7.30 (m, 1H), 7.06 (dd, *J* = 8.9, 2.5 Hz, 1H), 5.91 (ddt, *J* = 17.2, 10.7, 5.4 Hz, 1H), 5.30 (dq, *J* = 17.3, 1.7 Hz, 1H), 5.22 (dq, *J* = 10.5, 1.4 Hz, 1H), 5.16 (s, 2H), 4.64 (dt, *J* = 5.4, 1.5 Hz, 2H), 4.23 (s, 2H).
^{13}C NMR (101 MHz, DMSO-d_6) δ 166.95, 160.81 (C-2), 155.84, 145.11, 141.51, 136.96, 132.11, 128.42, 127.84, 127.75, 118.05, 114.16, 110.98, 104.19, 69.97, 65.44, 34.65.

ESI MS *m/z* (%): 324 (100) [M+H$^+$]

Allyl (*E*)-2-(5-(benzyloxy)benzo[*d*]oxazol-2-yl)-3-(dimethylamino)acrylate (**9b**)

The reaction was performed in the same manner as for **9a**, starting from **8b** (6.76 g, 20.9 mmol), affording **9b** as a brown oil (7.91 g, quantitative yield).

^{1}H NMR (401 MHz, $CDCl_3$) δ 7.80 (s, 1H), 7.45 (tt, *J* = 6.0, 1.5 Hz, 2H), 7.40 (d, *J* = 8.8 Hz, 1H), 7.42 – 7.36 (m, 2H), 7.36 – 7.29 (m, 1H), 7.27 (d, *J* = 2.6 Hz, 1H), 6.99 (dd, *J* = 8.8, 2.5 Hz, 1H), 5.88 (ddt, *J* = 17.2, 10.5, 5.3 Hz, 1H), 5.23 (dq, *J* = 17.2, 1.7 Hz, 1H) and 5.12 (dq, *J* = 10.5, 1.7 Hz, 1H), 5.09 (s, 1H), 4.63 (dt, *J* = 5.2, 1.6 Hz, 2H), 3.26 – 3.01 (m, 3H) and 2.71 – 2.43 (m, 3H).
^{13}C NMR (101 MHz, $CDCl_3$) δ 167.73, 161.87, 156.21, 154.43, 145.72, 142.32, 137.04, 133.07, 128.72, 128.13, 127.68, 116.96, 113.96, 110.80, 104.37, 86.16, 71.00, 64.75.

ESI MS *m/z* (%): 379 (100) [M+H$^+$]

Allyl 8-(benzyloxy)-1-oxo-2-phenyl-1*H*-benzo[4,5]oxazolo[3,2-*a*]pyridine-4-carboxylate (**10b**)

The reaction was performed in the same manner as for **10a**, starting from **9b** (7.90 g, 20.88 mmol), affording **10b** (4.48 g, 48%) as a yellow solid.

^{1}H NMR (401 MHz, $CDCl_3$) δ 8.40 (d, *J* = 2.6 Hz, 1H), 8.33 (s, 1H), 7.78 – 7.69 (m, 2H), 7.55 (d, *J* = 9.1 Hz, 1H), 7.50 – 7.32 (m, 8H), 7.16 (dd, *J* = 9.1, 2.7 Hz, 1H), 6.08 (ddt, *J* = 17.2, 10.4, 5.6 Hz, 1H), 5.49 (dq, *J* = 17.2, 1.6 Hz, 1H), 5.33 (dq, *J* = 10.4, 1.3 Hz, 1H), 5.16 (s, 2H), 4.90 (dt, *J* = 5.6, 1.5 Hz, 2H).
^{13}C NMR (101 MHz, $CDCl_3$) δ 162.82, 158.89, 156.78, 154.92, 141.66, 138.94, 136.26, 135.55, 132.29, 128.82, 128.75, 128.56, 128.39, 127.91, 127.9, 127.76, 122.67, 118.53, 116.11, 111.71, 102.69, 91.84, 71.14, 65.77.

ESI MS *m/z* (%): 452 (100) [M+H$^+$]

Allyl 8-hydroxy-1-oxo-2-phenyl-1*H*-benzo[4,5]oxazolo[3,2-*a*]pyridine-4-carboxylate (**11b**)

The reaction was performed in the same manner as for **11a**, starting from **10b** (1.88 g, 4.17 mmol), affording **11b** (1.51 g, quantitative yield) with free hydroxy group as a light-yellow solid, which was used in the next reaction without further purification.

ESI MS *m/z* (%): 362 (100) [M+H$^+$]

Allyl 8-(cyclohexyloxy)-1-oxo-2-phenyl-1*H*-benzo[4,5]oxazolo[3,2-*a*]pyridine-4-carboxylate (**12b**)

The reaction was performed in the same manner as for **12a**, starting from **11b** (1.50 g, 4.15 mmol), affording **12b** (1.37 g, 74%) as a yellow solid.

^{1}H NMR (401 MHz, $CDCl_3$) δ 8.32 (s, 1H), 8.29 (d, *J* = 2.6 Hz, 1H), 7.77 – 7.69 (m, 2H), 7.52 (d, *J* = 9.0 Hz, 1H), 7.49 – 7.42 (m, 2H), 7.40 – 7.33 (m, 1H), 7.07 (dd, *J* = 9.0, 2.6 Hz, 1H), 6.08 (ddt, *J* = 17.2, 10.4, 5.6 Hz, 2H), 5.49 (dq, *J* = 17.2, 1.6 Hz, 1H), 5.33 (dq, *J* = 10.4, 1.3 Hz, 1H), 4.89 (dt, *J* = 5.5, 1.5 Hz, 2H), 4.36 (tt, *J* = 8.5, 3.8 Hz, 1H), 2.05 – 1.51 (m, 10H).
^{13}C NMR (101 MHz, $CDCl_3$) δ 162.73, 158.82, 155.80, 154.83, 141.25, 138.80, 135.49, 132.18, 128.64, 128.44, 127.75, 122.51, 118.38, 117.18, 111.49, 103.86, 91.66, 76.78, 65.61, 31.61, 25.55, 25.38, 23.62.

ESI MS *m/z* (%): 444 (100) [M+H$^+$]

8-(Cyclohexyloxy)-1-oxo-2-phenyl-1*H*-benzo[4,5]oxazolo[3,2-*a*]pyridine-4-carboxylic acid (**14**)

12b (1.25 g, 2.82mmol, 1.0 equiv) was dissolved in ACN (28.0 mL) under Ar and cooled to 0 °C. Triethylsilane (538 8 μL, 3.38 mmol, 1.2 equiv) was added dropwise, followed by addition of $Pd(OAc)_2$ (63 mg, 0.28 mmol, 0.1 equiv) and PPh_3 (370 mg, 1.41 mmol, 0.5 equiv). The heterogeneous reaction mixture was stirred at 0 °C for 30 min, then at ambient temperature overnight. The reaction was treated with 0.1 M HCl solution (50.0 mL), stirred for 30 min, and the solution was extracted with EtOAc several times. The combined organic layers were washed with brine, dried with anhydrous Na_2SO_4 and concentrated under reduced pressure to give the product **14** as a light brown solid (1.13 g, quantitative yield).

^{1}H NMR (400 MHz, DMSO-d_6) δ 12.96 (s, 1H), 8.17 (s, 1H), 8.08 (d, J = 2.6 Hz, 1H), 7.79 (d, J = 9.1 Hz, 1H), 7.76-7.71 (m, 2H), 7.47-7.41 (m, 2H), 7.39 – 7.31 (m, 1H), 7.17 (dd, J = 9.0, 2.6 Hz, 1H), 4.40 (tt, J = 8.4, 3.7 Hz, 1H), 2.00 – 1.91 (m, 2H), 1.79 – 1.70 (m, 2H), 1.60 – 1.16 (m, 6H).
^{13}C NMR (101 MHz, DMSO-d_6) δ 163.80, 158.02, 154.71, 154.68, 141.09, 138.87, 135.58, 128.24, 128.22, 127.70, 127.33, 120.47, 115.70, 111.75, 103.64, 91.79, 75.88, 31.13, 25.07, 23.02.

ESI MS *m/z* (%): 404 (100) [M+H$^+$]

Methyl (8-(cyclohexyloxy)-1-oxo-2-phenyl-1*H*-benzo[4,5]oxazolo[3,2-*a*]pyridine-4-carbonyl)-L-tyrosinate (**15**)

To a solution of **14** (1.20 g, 2.97 mmol, 1.0 equiv) and L-tyrosine methyl ester (706 mg, 3.62 mmol, 1.2 equiv) in a DCM (50.0 mL)/DMF (50.0 mL) solvent mixture were sequentially added HOBt (493 mg, 3.65 mmol, 1.2 equiv), TEA (0.5 mL, 3.60 mmol, 1.2 equiv) and EDCI (692 mg, 3.61 mmol, 1.2 equiv), and the reaction mixture was stirred at ambient temperature for 12 h. The volatiles were evaporated and the residue was isolated by flash chromatography (EtOAc with 10% MeOH in cyclohexane, from 0% to 40%) to afford the product **15** (1.36 g, 79%) as a light-yellow solid.

^{1}H NMR (400 MHz, $CDCl_3$) δ 8.41 (s, 1H), 8.26 (d, J = 2.6 Hz, 1H), 7.76 – 7.68 (m, 2H), 7.45 – 7.39 (m, 2H), 7.36 (d, J = 9.1 Hz, 1H), 7.36-7.31 (m, 1H), 7.19 (d, J = 7.5 Hz, 1H), 7.07-7.03 (m, 2H), 7.04 (dd, J = 2.4, 9.2 Hz, 1H), 6.79 – 6.74 (m, 2H), 5.69 (d, J = 50.2 Hz, 1H), 5.11 (dt, J = 7.5, 5.6 Hz, 1H), 4.35 (td, J = 8.6, 4.2 Hz, 1H), 3.80 (s, 3H), 3.21 (dd, J = 5.6, 1.4 Hz, 2H), 2.03 – 1.44 (m, 10H).
^{13}C NMR (101 MHz, $CDCl_3$) δ 172.48, 161.69, 159.07, 156.08, 155.30, 152.31, 140.63, 138.90, 135.53, 130.70, 128.75, 128.50, 127.94, 127.67, 123.46, 117.04, 115.78, 111.13, 104.22, 93.76, 76.95, 53.82, 52.67, 37.18, 31.71, 25.66, 23.71.

ESI MS *m/z* (%): 581 (100) [M+H$^+$]

(8-(Cyclohexyloxy)-1-oxo-2-phenyl-1*H*-benzo[4,5]oxazolo[3,2-*a*]pyridine-4-carbonyl)-L-tyrosine (**2**) and (1-(5-(cyclohexyloxy)-2-hydroxyphenyl)-2-hydroxy-6-oxo-5-phenyl-1,6-dihydropyridine-3-carbonyl)-L-tyrosine (**16**)

A solution of the methyl ester **15** (679 mg, 1.17 mmol, 1.0 equiv) and aqueous 1 M LiOH solution (5.8 mL, 5.85 mmol, 5.0 equiv) in 1,4-dioxane (6.0 mL) was stirred for 45 min at ambient temperature. Then, the reaction mixture was poured into ice water, acidified with 1 M HCl to pH 3, and extracted with EtOAc three times. The combined organic phase was washed with brine, dried over anhydrous Na_2SO_4 and concentrated under reduced pressure. The product **2** (195 mg, 29%) and the ring-opened derivative (as mixture of tautomers) **16** (168 mg, 25%) were isolated by reversed phase flash column chromatography (ACN in H_2O, from 10% to 100%, 0.1% FA as modifier) as white solids.

Compound **2:** ^{1}H NMR (400 MHz, DMSO-d_6) δ 12.81 (s, 1H), 9.24 (s, 1H), 8.18 (s, 1H), 8.09 (d, J = 2.7 Hz, 1H), 8.04 (d, J = 7.7 Hz, 1H), 7.79 – 7.74 (m, 2H), 7.68 (d, J = 9.1 Hz, 1H), 7.46 (t, J = 7.6 Hz, 2H), 7.39 – 7.32 (m, 1H), 7.19 (dd, J = 9.1, 2.7 Hz, 1H), 7.17 – 7.13 (m, 2H), 6.72 – 6.67 (m, 2H), 4.66 – 4.56 (m, 1H), 4.41 (tt, J = 8.4, 3.7 Hz, 1H), 3.10 (dd, J = 13.9, 4.9 Hz, 1H), 3.01 (dd, J = 13.9, 8.9 Hz, 1H), 1.97 (d, J = 12.3 Hz, 2H), 1.81 – 1.67 (m, 2H), 1.61 – 1.25 (m, 6H).
^{13}C NMR (101 MHz, DMSO-d_6) δ 173.04, 161.57, 157.60, 156.04, 154.65, 152.77, 140.79, 137.82, 135.75, 130.22, 128.28, 128.16, 127.76, 127.47, 127.27, 120.35, 115.57, 115.11, 111.50, 103.80, 94.28, 75.91, 54.25, 35.76, 31.15, 25.07, 23.04.

ESI MS *m/z* (%): 567 (100) [M+H$^+$]
HRMS ESI ($C_{33}H_{29}O_7N_2$) calculated: 565.19802; found: 565.19788

Compound **16**: ^{1}H NMR (400 MHz, DMSO-d_6) δ 9.22 (s, 1H), 8.320 and 8.317 (s, 1H)^a, 7.66 (ddt, *J* = 7.7, 2.6, 1.4 Hz, 2H), 7.40 (ddd, *J* = 7.9, 7.0, 1.3 Hz, 2H), 7.31 – 7.25 (m, 1H), 7.13 – 7.06 (m, 2H), 6.88 – 6.78 (m, 2H), 6.76 and 6.75 (s, 1H)^a, 6.70 – 6.65 (m, 2H), 4.66 – 4.56 (m, 1H), 4.12 (tt, *J* = 8.1, 3.7 Hz, 1H), 3.13 (dd, *J* = 13.9, 4.8 Hz, 1H), 2.95 (dd, *J* = 14.0, 4.6 Hz, 1H), 1.87 (m, 2H), 1.72 – 1.65 (m, 2H), 1.53 – 1.17 (m, 6H).
^{13}C NMR (101 MHz, DMSO-d_6) δ 172.56, 169.56, 164.51, 160.59, 155.97, 149.91, 146.83 and 146.79^a, 136.97, 135.2, 129.92 and 129.90^a, 128.14 and 128.12^a, 127.86, 127.64, 126.56, 122.94, 118.0^a, 117.66, 117.13 and 117.07^a, 116.71 and 116.66^a, 115.19 and 115.15^a, 89.0^a, 75.17 and 75.12^a, 54.50 and 54.43^a, 35.47, 31.43, 25.19, 23.19.

ESI MS *m/z* (%): 585 (100) [M+H$^+$]
HRMS ESI ($C_{33}H_{31}O_8N_2$) calculated: 583.20859; found: 583.20807

^{a}The compound was measured as tautomeric mixture.

Methyl 5-bromo-1-methyl-6-oxo-1,6-dihydropyridine-3-carboxylate (**18**) [2]

To a solution of methyl 5-bromo-6-oxo-1,6-dihydropyridine-3-carboxylate (**17**, 500 mg, 2.16 mmol, 1.0 equiv) and K_2CO_3 (596 mg, 4.31 mmol, 2.0 equiv) in DMF (5.0 mL) was added MeI (201 µL, 3.23 mmol, 1.5 equiv), and the reaction mixture was stirred at ambient temperature for 2.5 h and was monitored by TLC (10% MeOH in DCM) and UPLC–MS. After completion, the reaction mixture was poured in water (15.0 mL), stirred for 10 min and extracted with EtOAc (3 × 13.0 mL). The combined organic phases were dried with anhydrous Na_2SO_4, filtered, and evaporated under reduced pressure to obtain **18** (459 mg, yield 87%) as an off-white solid. NMR spectra were consistent with the literature [3].

^{1}H NMR (401 MHz, $CDCl_3$) δ 8.26 (d, *J* = 2.3 Hz, 1H), 8.18 (d, *J* = 2.3 Hz, 1H), 3.87 (s, 3H), 3.66 (s, 3H).
^{13}C NMR (101 MHz, $CDCl_3$) δ 163.72, 159.29, 142.52, 140.48, 115.44, 109.80, 52.38, 39.53.

ESI MS *m/z* (%): 247 (100) [M+H$^+$]

5-Bromo-1-methyl-6-oxo-1,6-dihydropyridine-3-carboxylic acid (**19**) [2]

A solution of **18** (408 mg, 1.66 mmol, 1.0 equiv) and LiOH·H_2O (282 mg, 6.72 mmol, 4.1 equiv) in EtOH (7.0 mL)/H_2O (3.5 mL) solvent mixture was stirred at ambient temperature for 15 min, and the reaction was monitored by UPLC–MS. After completion, the mixture was concentrated to remove EtOH and acidified with aqueous HCl solution to pH 3. The acquired white precipitate was filtered, washed with ice-cold water and dried in vacuo to afford **19** as a white solid (384 mg, quantitative yield). NMR spectra were consistent with the literature [4].

^1H NMR (500 MHz, DMSO-d_6) δ 8.53 (d, J = 2.3 Hz, 1H), 8.15 (d, J = 2.3 Hz, 1H), 3.57 (s, 3H).
^{13}C NMR (126 MHz, DMSO-d_6) δ 164.49, 158.54, 144.37, 140.51, 113.38, 109.54, 38.74.

ESI MS *m/z* (%): 233 (100) [M+H$^+$]

Methyl (5-bromo-1-methyl-6-oxo-1,6-dihydropyridine-3-carbonyl)-L-tyrosinate (**20**)

EDCI (352 mg, 1.84 mmol, 1.1 equiv) was added to a solution of **19** (380 mg, 1.64 mmol, 1.0 equiv), HOBt (256 mg, 1.90 mmol, 1.2 equiv), L-tyrosine methyl ester (356 mg, 1.82 mmol, 1.1 equiv) and TEA (700 µL, 5.03 mmol, 3.1 equiv) in DCM (6.0 mL)/DMF (6.0 mL) solvent mixture, and the cloudy solution was stirred overnight at ambient temperature. Then, the solvents were evaporated, the residue was diluted with water (30.0 mL), acidified with 1 M HCl (pH 5–6), and extracted with DCM (3 × 30.0 mL). The combined organic phases were washed with brine, dried with anhydrous Na_2SO_4, filtered, and the solvent was evaporated to yield the product **20** as a yellow oil (670 mg, quantitative yield).

^1H NMR (400 MHz, DMSO-d_6) δ 9.21 (s, 1H), 8.64 (d, J = 7.7 Hz, 1H), 8.40 (d, J = 2.4 Hz, 1H), 8.36 (d, J = 2.4 Hz, 1H), 7.06 – 7.00 (m, 2H), 6.68 – 6.62 (m, 2H), 4.54 (ddd, J = 9.6, 7.6, 5.6 Hz, 1H), 3.61 (s, 3H), 3.55 (s, 3H), 3.00 (dd, J = 13.9, 5.7 Hz, 1H), 2.94 – 2.87 (dd, J = 13.9, 9.6 Hz, 1H).
^{13}C NMR (101 MHz, DMSO-d_6) δ 172.14, 162.49, 158.15, 155.96, 142.35, 139.27, 129.91, 127.35, 115.07, 113.23, 111.67, 54.59, 51.88, 38.75, 35.64.

ESI MS *m/z* (%): 411 (100) [M+H$^+$]

(1-Methyl-6-oxo-5-phenyl-1,6-dihydropyridine-3-carbonyl)-L-tyrosine (**3a**)

20 (99 mg, 0.18 mmol, 1.0 equiv), phenylboronic acid (34 mg, 0.28 mmol, 1.5 equiv), Pd(dppf)$Cl_2 \cdot CH_2Cl_2$ (15 mg, 0.02 mmol, 0.1 equiv) and Cs_2CO_3 (255 mg, 0.78 mmol, 4.3 equiv) were dissolved in DMF (2.0 mL)/H_2O (0.5 mL) solvent mixture, and the dark red solution was stirred at 80 °C overnight under argon atmosphere. The reaction mixture was acidified with excess of formic acid and concentrated under reduced pressure. The residue was purified by flash column chromatography (EtOAc/acetone/EtOH/H_2O, 17:3:3:2 mixture in EtOAc, from 10% to 100%), followed by reversed phase flash column chromatography (MeOH in H_2O, from 10% to 100%) to yield the final product **3a** as a white solid (18 mg, 24%).

^1H NMR (401 MHz, MeOD) δ 8.19 (d, J = 2.6 Hz, 1H), 7.95 (d, J = 2.6 Hz, 1H), 7.66 – 7.60 (m, 3H), 7.44 – 7.37 (m, 2H), 7.37 – 7.31 (m, 1H), 7.11 – 7.06 (m, 2H), 6.73 – 6.66 (m, 2H), 4.77 (dd, J = 9.6, 5.1 Hz, 1H), 3.64 (s, 3H), 3.23 (dd, J = 14.0, 5.1 Hz, 1H), 2.99 (dd, J = 14.0, 9.6 Hz, 1H).
^{13}C NMR (101 MHz, MeOD) δ 175.05, 166.68, 163.65, 157.34, 141.89, 137.58, 137.41, 131.22, 131.15, 129.76, 129.32, 129.21, 129.09, 116.24, 114.84, 55.89, 39.20, 37.46.

ESI MS *m/z* (%): 393 (100) [M+H$^+$]
HRMS ESI ($C_{22}H_{19}O_5N_2$) calculated: 391.12994; found: 391.12976

<u>(1-Methyl-2-oxo-1,2-dihydro-[3,3'-bipyridine]-5-carbonyl)-L-tyrosine (**3b**)</u>

20 (87 mg, 0.16 mmol, 1.0 equiv), 3-pyridinylboronic acid (34 mg, 0.27 mmol, 1.7 equiv), Pd(dppf)Cl_2·CH_2Cl_2 (16 mg, 0.02 mmol, 0.1 equiv) and Cs_2CO_3 (248 mg, 0.76 mmol, 4.7 equiv) were dissolved in DMF (2.5 mL)/H_2O (0.75 mL) solvent mixture, and the dark red solution was stirred at 80 °C overnight under argon atmosphere. The reaction mixture was treated with excess of formic acid and concentrated under reduced pressure. The residue was purified by normal phase flash column chromatography (EtOAc/acetone/EtOH/H_2O, 17:3:3:2 mixture in EtOAc, from 10% to 100%), followed by reversed phase flash column chromatography (ACN in H_2O, from 10% to 100%, 0.1% FA as modifier) to yield the final product **3b** as an off-white solid (15 mg, 24%).

^{1}H NMR (400 MHz, MeOD) δ 8.85 (s, 1H), 8.49 (m, 1H), 8.38 (s, 1H), 8.30 (d, J = 2.6 Hz, 1H), 8.15 (dt, J = 8.1, 1.8 Hz, 1H), 8.06 (d, J = 2.5 Hz, 1H), 7.48 (dd, J = 8.0, 4.8 Hz, 1H), 7.12 – 7.06 (m, 2H), 6.69 – 6.64 (m, 2H), 4.71 (dd, J = 8.8, 4.8 Hz, 1H), 3.64 (s, 3H), 3.23 (dd, J = 14.1, 4.9 Hz, 1H), 2.99 (dd, J = 14.0, 8.6 Hz, 1H).
^{13}C NMR (101 MHz, MeOD) δ 170.44, 165.82, 163.19, 157.10, 149.73, 149.04, 142.94, 138.27, 138.09, 134.1, 131.36, 130.03, 127.00, 124.87, 116.07, 115.10, 57.38, 39.19, 38.07.

ESI MS *m/z* (%): 394 (100) [M+H$^+$]
HRMS ESI ($C_{21}H_{18}O_5N_3$) calculated: 392.12519; found: 392.12537

<u>(1-Methyl-6-oxo-5-(thiophen-3-yl)-1,6-dihydropyridine-3-carbonyl)-L-tyrosine (**3c**)</u>

20 (87 mg, 0.16 mmol, 1.0 equiv), 3-thienylboronic acid (35 mg, 0.28 mmol, 1.7 equiv), Pd(dppf)Cl_2·CH_2Cl_2 (15 mg, 0.02 mmol, 0.1 equiv) and Cs_2CO_3 (242 mg, 0.74 mmol, 4.6 equiv) were dissolved in DMF (2.5 mL)/H_2O (0.75 mL) solvent mixture, and the dark red solution was stirred at 80 °C overnight under argon atmosphere. The reaction mixture was acidified with excess formic acid and concentrated under reduced pressure. The residue was purified by reversed phase flash column chromatography (ACN in H_2O, to 10% to 100%, 0.1% FA as modifier), followed by normal phase flash column chromatography (EtOAc/acetone/EtOH/H_2O, 17:3:3:2 mixture in EtOAc, from 10% to 100%) to yield the pure final product **3c** as an pale brown solid (17 mg, 26%).

^{1}H NMR (400 MHz, MeOD) δ 8.16 (dd, J = 3.0, 1.3 Hz, 1H), 8.15 (d, J = 2.5 Hz, 1H), 8.12 (d, J = 2.6 Hz, 1H), 7.55 (dd, J = 5.1, 1.3 Hz, 1H), 7.42 (dd, J = 5.1, 3.1 Hz, 1H), 7.13 – 7.08 (m, 2H), 6.73 – 6.68 (m, 2H), 4.77 (dd, J = 9.2, 5.0 Hz, 1H), 3.61 (s, 3H), 3.25 (dd, J = 14.0, 5.0 Hz, 1H), 3.01 (dd, J = 14.0, 9.2 Hz, 1H).
^{13}C NMR (101 MHz, MeOD) δ 176.01, 166.50, 163.01, 157.24, 140.77, 137.27, 135.10, 131.30, 129.61, 127.86, 125.93, 125.64, 125.51, 116.20, 114.68, 56.45, 39.20, 37.71.

ESI MS *m/z* (%): 399 (100) [M+H$^+$]
HRMS ESI ($C_{20}H_{17}O_5N_2S$) calculated: 397.08637; found: 397.08610

2-Phenylthiazolo[2,3-*b*]thiazol-4-ium-3-olate (**22**) [5]

A solution of 2-bromo-2-phenylacetyl chloride (prepared according to a published procedure [6,7], 4.00 g, 17.07 mmol, 1.0 equiv) in dry THF (10.0 mL) was added dropwise to a suspension of 1,3-thiazole-2-thiol (**21**, 2.00 g, 17.07 mmol, 1.0 equiv) in dry THF (10.0 mL). After 5 min, TEA (5.5 mL, 39.26 mmol, 2.3 equiv) was added slowly, and the reaction mixture was stirred for 1 h. The formed yellow crystalline product was filtered and washed with water, followed by diethyl ether. Trituration with chloroform/hexane mixture afforded the reactive compound **22** as a deep yellow powder (1.74 g, 44%), which was used in the next reaction without further purification.

ESI MS *m/z* (%): 234 (100) [M+H$^+$]

2-Methyl-6-phenylthiazolo[2,3-*b*][1,3,4]thiadiazol-4-ium-5-olate (**27**) [5]

A solution of 2-bromo-2-phenylacetyl chloride (prepared according to a published procedure [6,7], 3.89 g, 16.64 mmol, 1.1 equiv) in dry THF (10.0 mL) was added dropwise to a suspension of 5-methyl-1,3,4-thiadiazole-2-thiol (**26**, 2.00 g, 15.13 mmol, 1.0 equiv) in dry THF (10.0 mL). After 5 min, TEA (5.3 mL, 37.82 mmol, 2.5 equiv) was added slowly at room temperature, and the reaction mixture was stirred for 20 min. The formed brown crystalline product was filtered and washed with water, followed by diethyl ether, affording the reactive compound **27** as a brown powder (2.88 g, 77%), which was used in the next reaction without further purification.

ESI MS *m/z* (%): 249 (8) [M+H$^+$], 281 (100) [M+MeOH+H$^+$]

Ethyl 5-oxo-6-phenyl-5*H*-thiazolo[3,2-*a*]pyridine-8-carboxylate (**23**) [5]

The mesoionic compound **22** (1.34 g, 5.75 mmol, 1.0 equiv) and ethyl acrylate (626 µL, 5.75 mmol, 1.0 equiv) in dry toluene (25.0 mL) were heated under reflux for 24 h, and the reaction was being monitored by TLC (5% MeOH in DCM). Toluene was removed under reduced pressure, leaving a dark colored residue that was purified by normal phase (MeOH in DCM, from 0% to 20%), and then reversed phase flash column chromatography (ACN in water, from 10% to 100%, 0.1% FA as a modifier) to obtain the product **23** as a pale brown solid (652 mg, 38%).

^{1}H NMR (400 MHz, $CDCl_3$) δ 8.39 (d, *J* = 4.5 Hz, 1H), 8.30 (s, 1H), 7.82 – 7.72 (m, 2H), 7.50 – 7.40 (m, 2H), 7.39 – 7.31 (m, 1H), 7.19 (d, *J* = 4.5 Hz, 1H), 4.44 (q, *J* = 7.1 Hz, 2H), 1.43 (t, *J* = 7.1 Hz, 3H).
^{13}C NMR (101 MHz, $CDCl_3$) δ 165.02, 158.73, 152.07, 136.57, 136.23, 128.77, 128.51, 127.74, 125.29, 120.69, 115.35, 103.21, 61.51, 14.64.

ESI MS *m/z* (%): 300 (100) [M+H$^+$]

Ethyl 2-methyl-5-oxo-6-phenyl-5*H*-[1,3,4]thiadiazolo[3,2-*a*]pyridine-8-carboxylate (**28**) [5]

The reaction was performed in the same manner as for **23**, starting from **27** (2.48 g, 10.00 mmol), affording **28** as a yellow solid (321 mg, 10%).

^{1}H NMR (400 MHz, $CDCl_3$) δ 8.19 (s, 1H), 7.79 – 7.74 (m, 2H), 7.49 – 7.40 (m, 2H), 7.40 – 7.31 (m, 1H), 4.42 (q, *J* = 7.1 Hz, 2H), 2.79 (s, 3H), 1.41 (t, *J* = 7.2 Hz, 3H).
^{13}C NMR (101 MHz, $CDCl_3$) δ 164.56, 160.74, 157.69, 150.63, 135.93, 135.50, 128.78, 128.50, 128.06, 124.75, 102.00, 61.92, 16.35, 14.56.

ESI MS *m/z* (%): 315 (100) [M+H$^+$]

5-Oxo-6-phenyl-5*H*-thiazolo[3,2-*a*]pyridine-8-carboxylic acid (**24**)

To a solution of **23** (300 mg, 1.00 mmol, 1.0 equiv) in MeOH (4.0 mL) was added NaOH (241 mg, 6.00 mmol, 6.0 equiv) dissolved in water (4.0 mL), and the reaction mixture was stirred at reflux for 2 h. After cooling down to ambient temperature, the pH of the mixture was adjusted to 1 with 1 M HCl solution, extracted with chloroform. The combined organic phases were dried with anhydrous Na_2SO_4 and concentrated under reduced pressure. The residual solid was dissolved in a minimal amount of DCM, and the product **24** was precipitated with MeOH as light brown needles (143 mg, 53%).

^{1}H NMR (400 MHz, DMSO-d_6) δ 8.38 (d, J = 4.5 Hz, 1H), 8.18 (s, 1H), 7.77 – 7.74 (m, 2H), 7.74 (s, 1H), 7.48 – 7.39 (m, 2H), 7.38 – 7.29 (m, 1H).
^{13}C NMR (101 MHz, DMSO-d_6) δ 165.75, 157.64, 152.16, 136.14, 136.02, 128.27, 128.19, 127.22, 124.71, 118.45, 117.58, 102.75.

ESI MS *m/z* (%): 272 (100) [M+H$^+$]

2-Methyl-5-oxo-6-phenyl-5*H*-[1,3,4]thiadiazolo[3,2-*a*]pyridine-8-carboxylic acid (**29**)

The reaction was performed in the same manner as for **24**, starting from **28** (100 mg, 0.32 mmol), affording **29** as pale yellow flakes (75 mg, 82%).

^{1}H NMR (400 MHz, DMSO-d_6) δ 8.09 (s, 1H), 7.76 – 7.68 (m, 2H), 7.44 (dd, J = 8.4, 6.8 Hz, 2H), 7.39 – 7.32 (m, 1H), 2.76 (s, 3H).
^{13}C NMR (101 MHz, DMSO-d_6) δ 165.45, 161.66, 156.57, 151.27, 136.01, 135.26, 128.39, 128.20, 127.53, 122.45, 101.54, 15.79.

ESI MS *m/z* (%): 287 (100) [M+H$^+$], 309 (18) [M+Na$^+$]

Methyl (5-oxo-6-phenyl-5*H*-thiazolo[3,2-*a*]pyridine-8-carbonyl)-L-tyrosinate (**25**)

To a solution of **24** (103 mg, 0.38 mmol, 1.0 equiv) and L-tyrosine methyl ester (89 mg, 0.46 mmol, 1.2 equiv) in DCM (6.0 mL)/DMF (6.0 mL) solvent mixture were sequentially added HOBt (62 mg, 0.46 mmol, 1.2 equiv), TEA (64 µL, 0.46 mmol, 1.2 equiv) and EDCI (88 mg, 0.46 mmol, 1.2 equiv), and the reaction mixture was stirred at ambient temperature for 12 h. The volatiles were evaporated, and the residue was isolated by reversed phase flash column chromatography (ACN in water, from 10% to 100%, 0.1% FA as a modifier) to afford the product **25** (40 mg, 23%) as a white solid.

^{1}H NMR (401 MHz, DMSO-d_6) δ 9.23 (s, 1H), 8.92 (d, J = 7.8 Hz, 1H), 8.57 (s, 1H), 8.33 (d, J = 4.5 Hz, 1H), 7.87 – 7.78 (m, 2H), 7.67 (d, J = 4.5 Hz, 1H), 7.47 (dd, J = 8.4, 7.0 Hz, 2H), 7.40 – 7.32 (m, 1H), 7.12 – 7.04 (m, 2H), 6.68 – 6.59 (m, 2H), 4.63 (ddd, J = 9.6, 7.7, 5.7 Hz, 1H), 3.63 (s, 3H), 3.06 (dd, J = 13.8, 5.7 Hz, 1H), 2.97 (dd, J = 13.8, 9.7 Hz, 1H).
^{13}C NMR (101 MHz, DMSO-d_6) δ 172.41, 163.98, 157.41, 155.97, 150.72, 136.39, 133.64, 130.01, 128.55, 128.09, 127.54, 127.16, 124.13, 118.10, 118.06, 115.10, 113.05, 104.71, 54.65, 51.98, 35.76.

ESI MS *m/z* (%): 449 (100) [M+H$^+$]

Methyl (2-methyl-5-oxo-6-phenyl-5*H*-[1,3,4]thiadiazolo[3,2-*a*]pyridine-8-carbonyl)-L-tyrosinate (**30**)

To a solution of **29** (55 mg, 0.19 mmol, 1.0 equiv) and L-tyrosine methyl ester (45 mg, 0.23 mmol, 1.2 equiv) in DCM (3.0 mL)/DMF (3.0 mL) solvent mixture were sequentially added HOBt (31 mg, 0.23 mmol, 1.2 equiv), TEA (32 µL, 0.23 mmol, 1.2 equiv) and EDCI (44 mg, 0.23 mmol, 1.2 equiv) and the reaction mixture was stirred at ambient temperature for 12 h. The volatiles were evaporated and the residue was isolated by reversed phase flash column chromatography (ACN in water, from 10% to 100%, 0.1% FA as a modifier) to afford the product **30** (20 mg, 22%) as a white solid.

^{1}H NMR (401 MHz, DMSO-d_6) δ 9.22 (s, 1H), 9.01 (d, *J* = 7.8 Hz, 1H), 8.54 (s, 1H), 7.82 – 7.75 (m, 2H), 7.48 (dd, *J* = 8.4, 6.9 Hz, 2H), 7.41 – 7.36 (m, 1H), 7.10 – 7.02 (m, 2H), 6.67 – 6.62 (m, 2H), 4.63 (ddd, *J* = 9.6, 7.7, 5.7 Hz, 1H), 3.62 (s, 3H), 3.05 (dd, *J* = 13.8, 5.8 Hz, 1H), 2.96 (dd, *J* = 13.8, 9.6 Hz, 1H), 2.70 (s, 3H).
^{13}C NMR (101 MHz, DMSO-d_6) δ 172.17, 163.21, 162.24, 156.38, 155.96, 149.49, 136.24, 133.10, 129.95, 128.63, 128.07, 127.46, 127.36, 122.22, 115.08, 103.31, 54.80, 51.96, 35.69, 15.61.

ESI MS *m/z* (%): 464 (100) [M+H$^+$], 486 (14) [M+Na$^+$]

(5-Oxo-6-phenyl-5H-thiazolo[3,2-a]pyridine-8-carbonyl)-L-tyrosine (**4a**)

A solution of the methyl ester **25** (20 mg, 0.05 mmol, 1.0 equiv) and aqueous 1 M LiOH solution (223 µL, 0.22 mmol, 5.0 equiv) in 1,4-dioxane (0.5 mL) was stirred for 2 h at ambient temperature. Then, the reaction mixture was poured into ice water, acidified with 1 M HCl to pH 3 and extracted with EtOAc three times. The combined organic phases were washed with brine, dried with anhydrous Na_2SO_4 and concentrated under reduced pressure. **4a** was isolated by reversed phase flash column chromatography (ACN in water, from 10% to 80%, 0.1% FA as a modifier) as a white solid (12 mg, 62%).

^{1}H NMR (600 MHz, DMSO-d_6) δ 9.19 (s, 1H), 8.81 (d, *J* = 8.2 Hz, 1H), 8.57 (s, 1H), 8.33 (d, *J* = 4.5 Hz, 1H), 7.86 – 7.82 (m, 2H), 7.66 (d, *J* = 4.5 Hz, 1H), 7.50 – 7.45 (m, 2H), 7.38 – 7.34 (m, 1H), 7.11 – 7.07 (m, 2H), 6.66 – 6.60 (m, 2H), 4.58 (ddd, *J* = 10.4, 8.1, 4.8 Hz, 1H), 3.08 (dd, *J* = 14.0, 4.8 Hz, 1H), 2.94 (dd, *J* = 14.0, 10.3 Hz, 1H).
^{13}C NMR (151 MHz, DMSO-d_6) δ 173.40, 163.96, 157.42, 155.87, 150.61, 136.42, 133.63, 130.00, 128.56, 127.15, 124.11, 118.14, 118.01, 115.05, 114.87, 104.97, 54.61, 40.43, 35.72.

ESI MS *m/z* (%): 435 (100) [M+H$^+$]
HRMS ESI ($C_{23}H_{17}O_5N_2S$) calculated: 433.08637; found: 433.08603

(2-Methyl-5-oxo-6-phenyl-5*H*-[1,3,4]thiadiazolo[3,2-*a*]pyridine-8-carbonyl)-L-tyrosine (**4b**)

A solution of the methyl ester **30** (10 mg, 0.02 mmol, 1.0 equiv) and aqueous 1 M LiOH solution (108 µL, 0.11 mmol, 5.0 equiv) in 1,4-dioxane (0.5 mL) was stirred for 2 h at ambient temperature. Then, the reaction mixture was poured into ice water, acidified with 1 M HCl to pH 3 and extracted with EtOAc three times. The combined organic phase was washed with brine, dried with anhydrous Na_2SO_4 and concentrated under reduced pressure. The product **4b** was isolated by reversed phase flash column chromatography (ACN in water, from 10% to 80%, 0.1% FA as a modifier) as a white solid (5 mg, 53%).

^{1}H NMR (600 MHz, DMSO-d_6) δ 9.19 (s, 1H), 8.90 (d, J = 8.2 Hz, 1H), 8.54 (s, 1H), 7.81 – 7.76 (m, 2H), 7.50 – 7.46 (m, 2H), 7.41 – 7.36 (m, 1H), 7.10 – 7.06 (m, 2H), 6.64 – 6.61 (m, 2H), 4.58 (ddd, J = 10.4, 8.1, 4.8 Hz, 1H), 3.08 (dd, J = 14.0, 4.8 Hz, 1H), 2.93 (dd, J = 14.0, 10.4 Hz, 1H), 2.70 (s, 3H).
^{13}C NMR (151 MHz, DMSO-d_6) δ 173.19, 163.19, 162.33, 156.44, 155.87, 149.37, 136.29, 133.13, 129.96, 128.67, 128.10, 127.96, 127.48, 122.24, 115.06, 103.59, 54.77, 35.70, 15.64.

ESI MS *m/z* (%): 450 (100) [M+H^{+}]
HRMS ESI ($C_{23}H_{18}O_5N_3S$) calculated: 448.09726; found: 448.09720

^{1}H NMR, ^{13}C NMR, HRMS spectra and UPLC–MS traces of the final compounds

(8-(Cyclohexyloxy)-1-oxo-2-phenyl-1*H*-benzo[4,5]oxazolo[3,2-*a*]pyridine-4-carbonyl)-L-tyrosine (**2**)

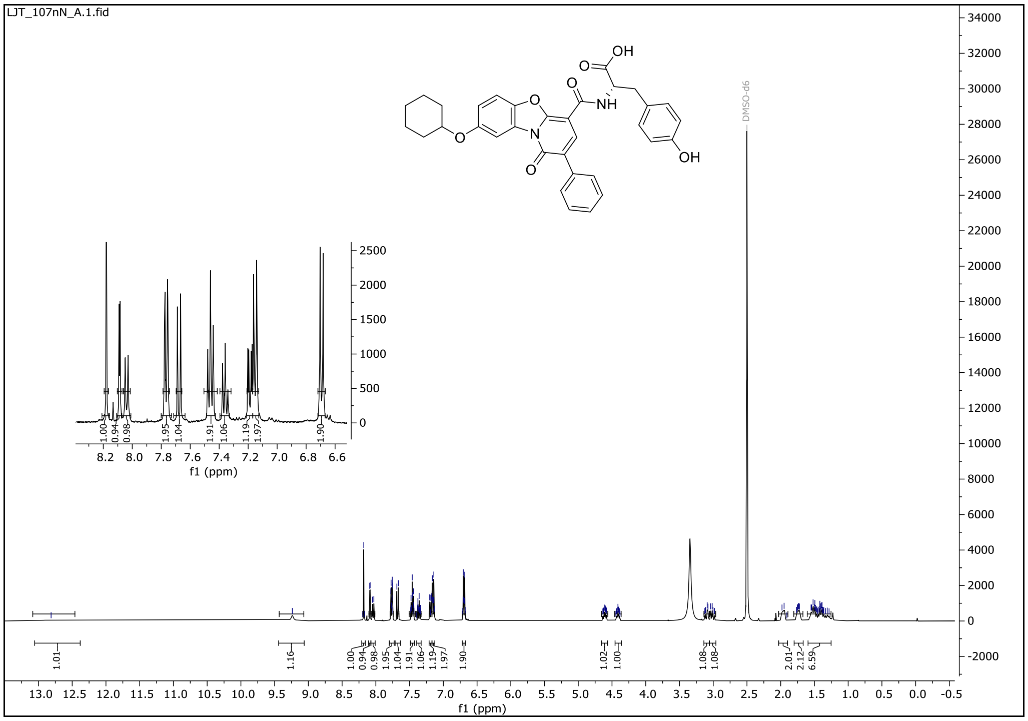

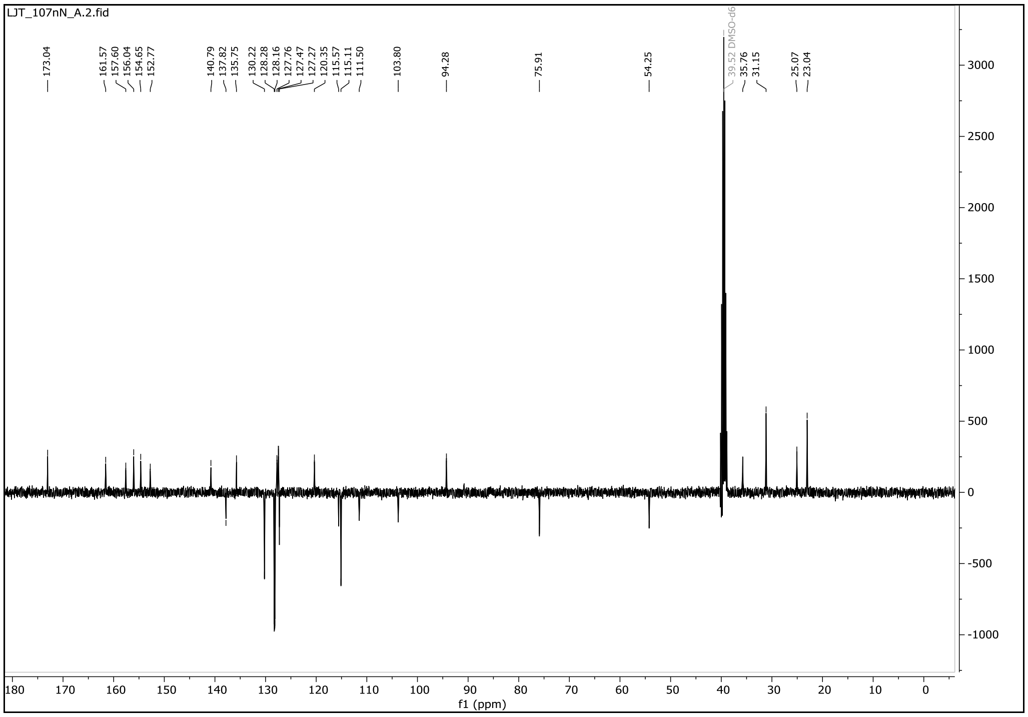

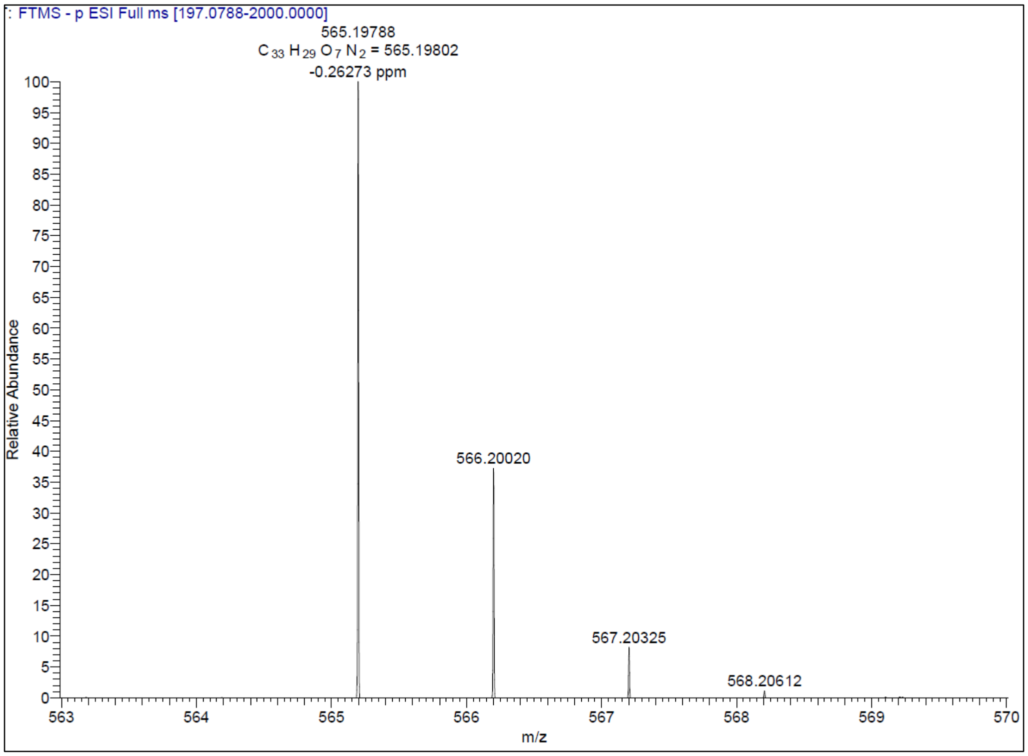
FTMS - p ESI Full ms [197.0788-2000.0000]
565.19788
C 33 H 29 O 7 N 2 = 565.19802
-0.26273 ppm
566.20020
567.20325
568.20612
Relative Abundance
m/z

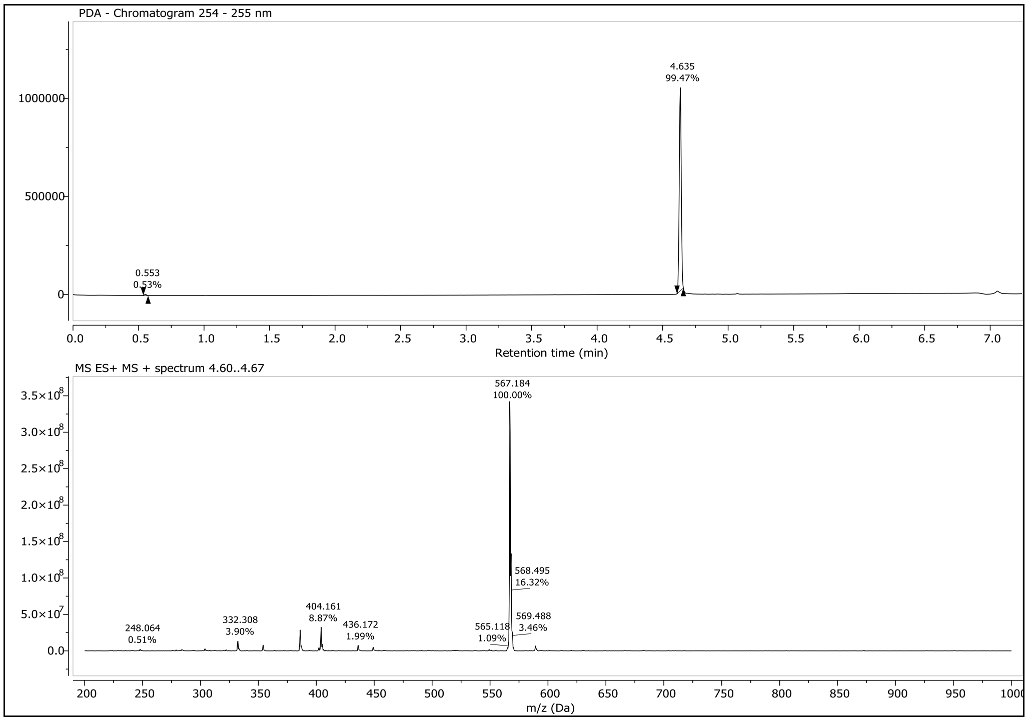
PDA - Chromatogram 254 - 255 nm
4.635
99.47%
0.553
0.53%
Retention time (min)
MS ES+ MS + spectrum 4.60..4.67
567.184
100.00%
568.495
16.32%
569.488
3.46%
565.118
1.09%
404.161
8.87%
436.172
1.99%
332.308
3.90%
248.064
0.51%
m/z (Da)

(1-(5-(Cyclohexyloxy)-2-hydroxyphenyl)-2-hydroxy-6-oxo-5-phenyl-1,6-dihydropyridine-3-carbonyl)-L-tyrosine (**16**)

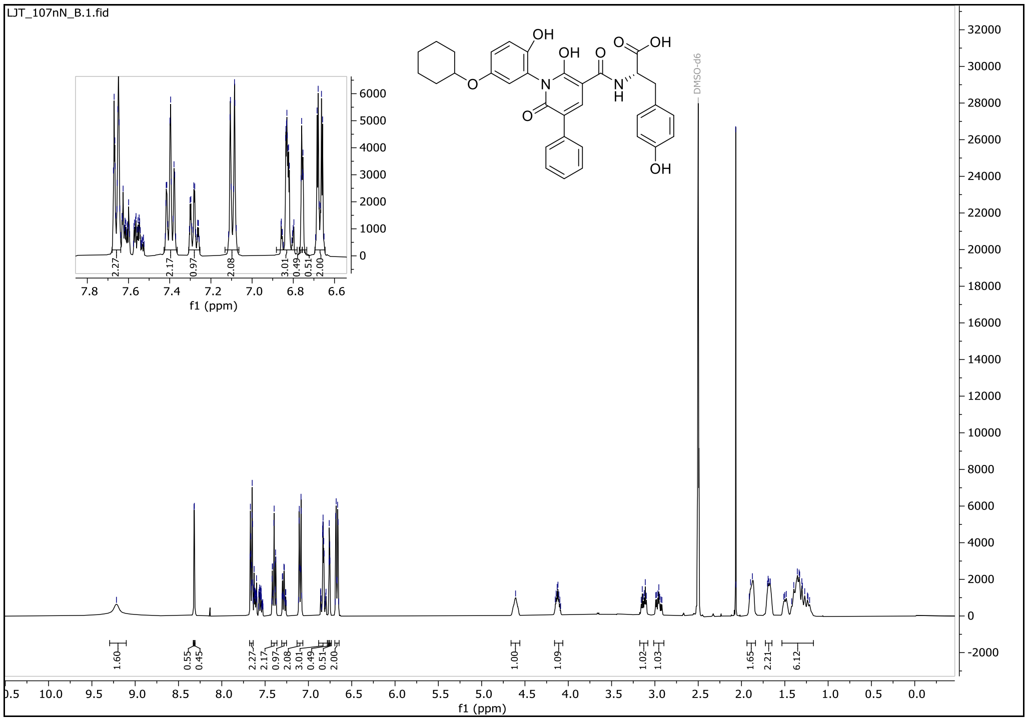

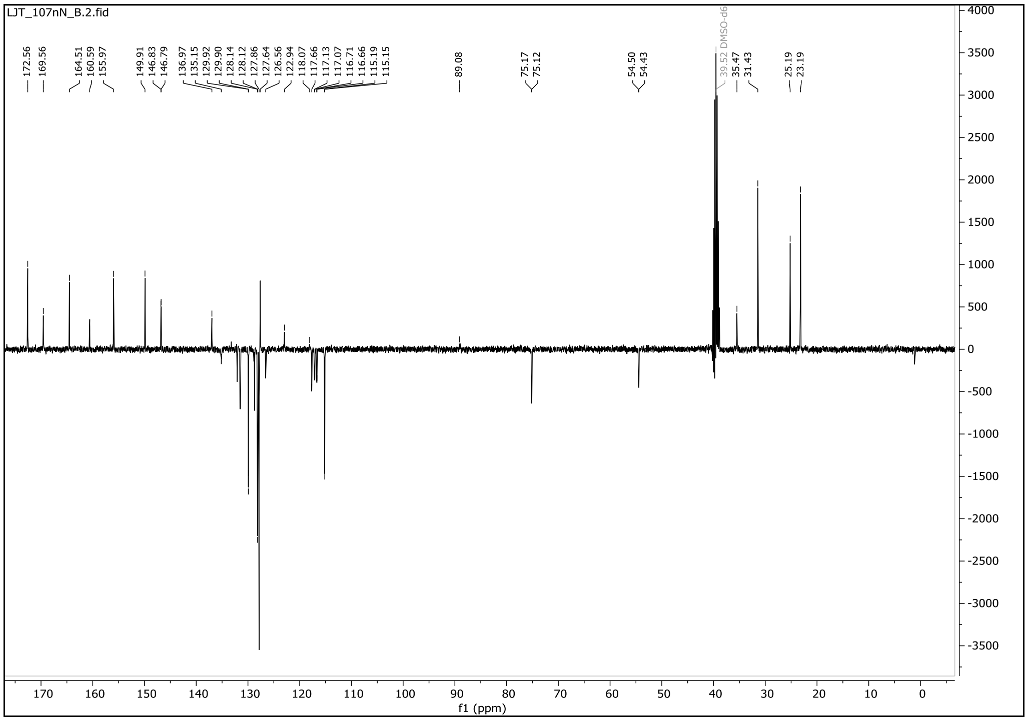

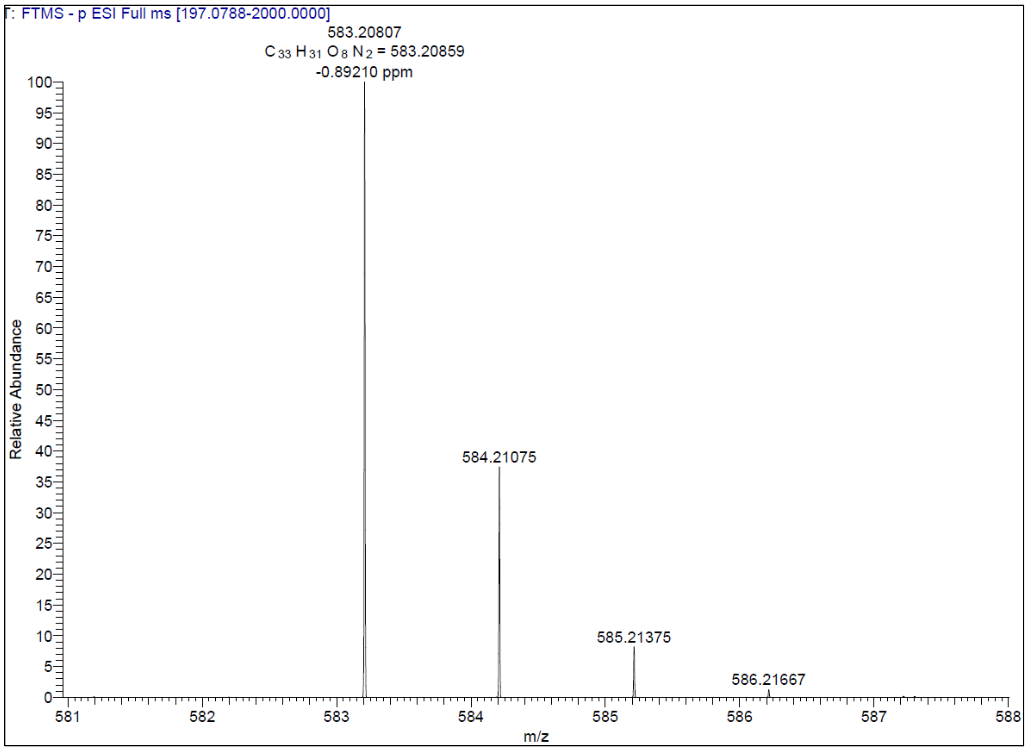
T: FTMS - p ESI Full ms [197.0788-2000.0000]
583.20807
$C_{33}H_{31}O_8N_2$ = 583.20859
-0.89210 ppm
584.21075
585.21375
586.21667
Relative Abundance
m/z

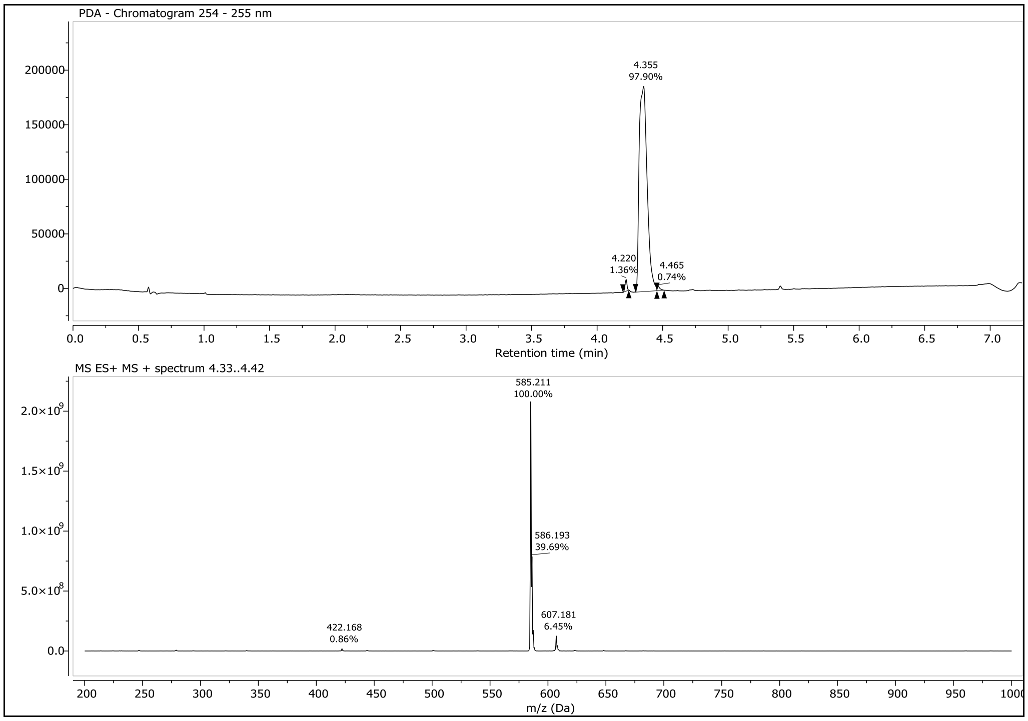
PDA - Chromatogram 254 - 255 nm
4.355
97.90%
4.220
1.36%
4.465
0.74%
Retention time (min)
MS ES+ MS + spectrum 4.33..4.42
585.211
100.00%
586.193
39.69%
607.181
6.45%
422.168
0.86%
m/z (Da)

(1-Methyl-6-oxo-5-phenyl-1,6-dihydropyridine-3-carbonyl)-L-tyrosine (**3a**)

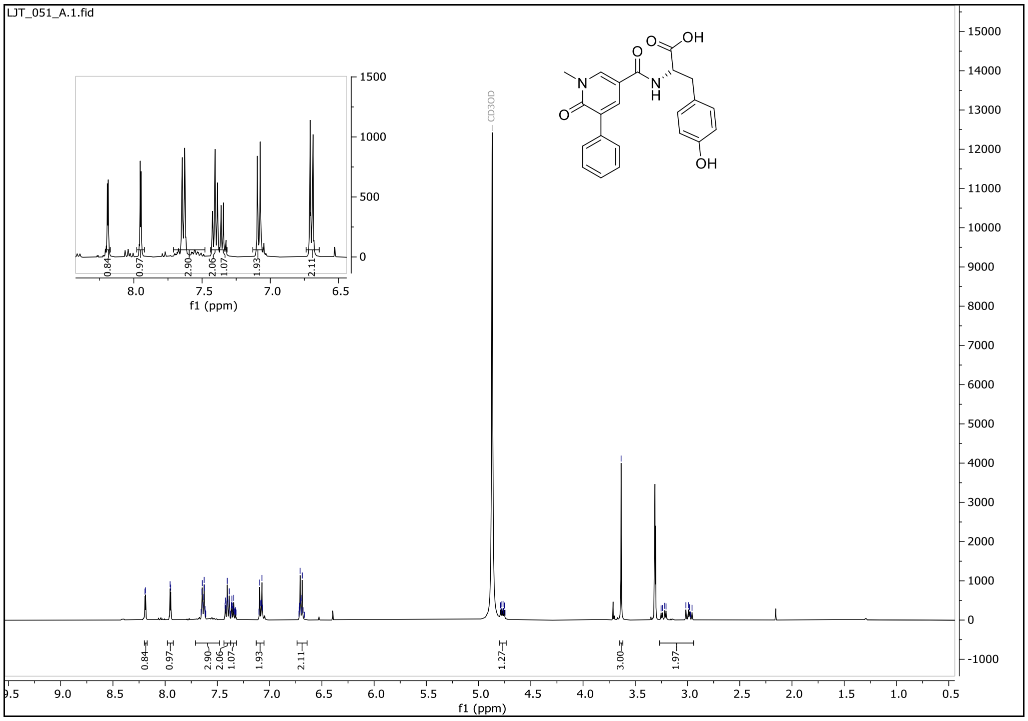

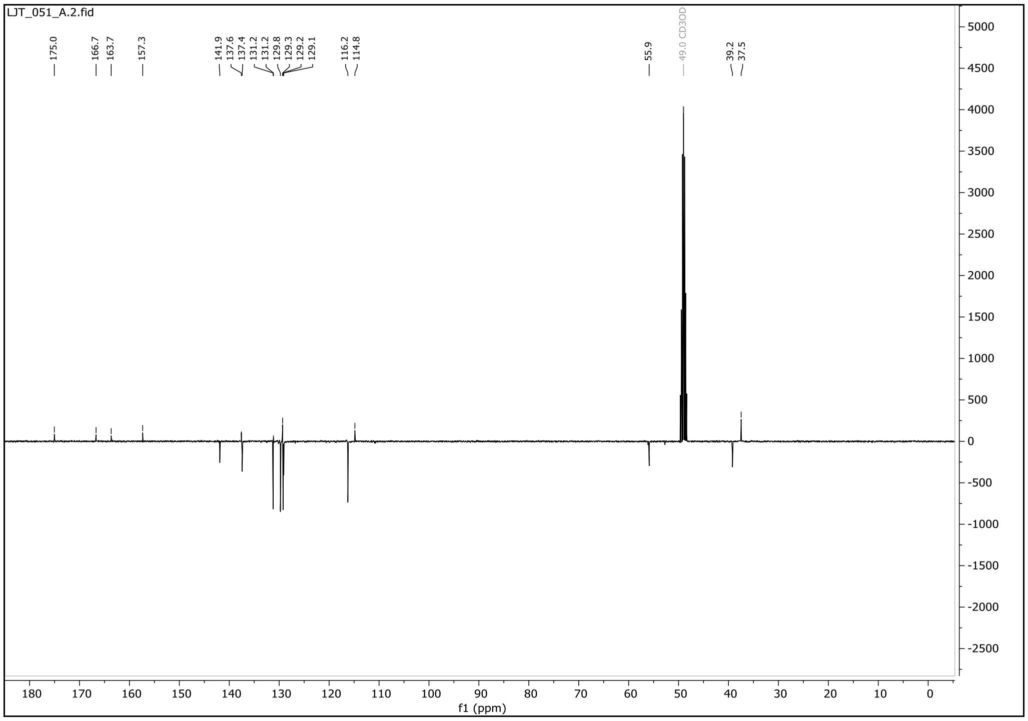

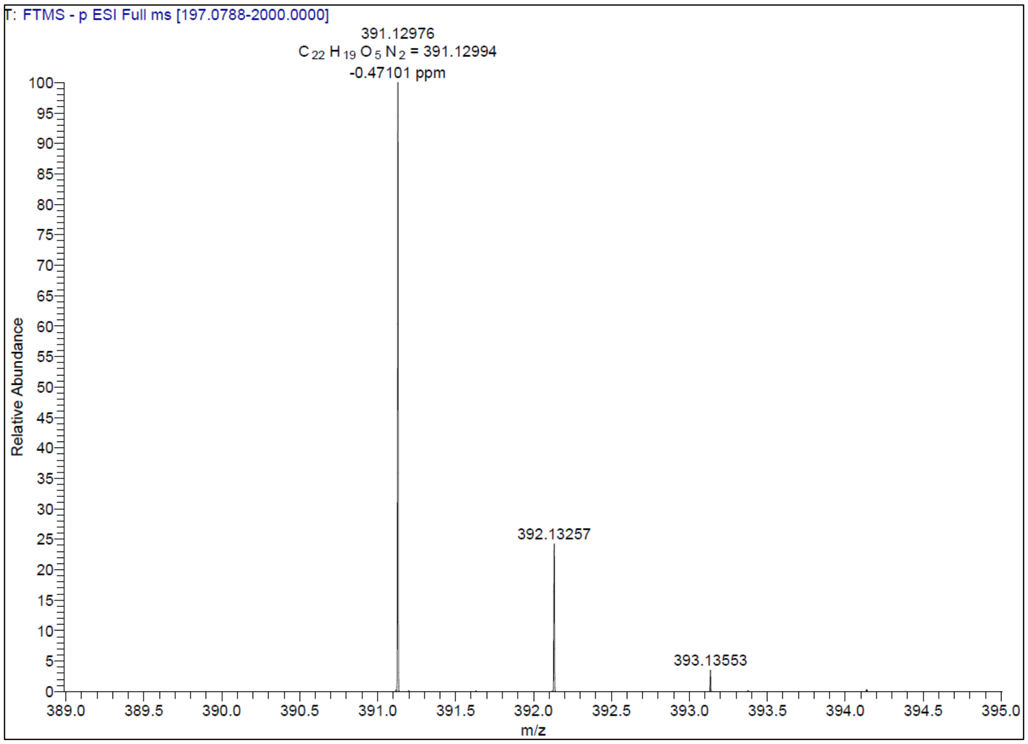
T: FTMS - p ESI Full ms [197.0788-2000.0000]
391.12976
$C_{22}H_{19}O_5N_2$ = 391.12994
-0.47101 ppm
392.13257
393.13553
Relative Abundance
m/z

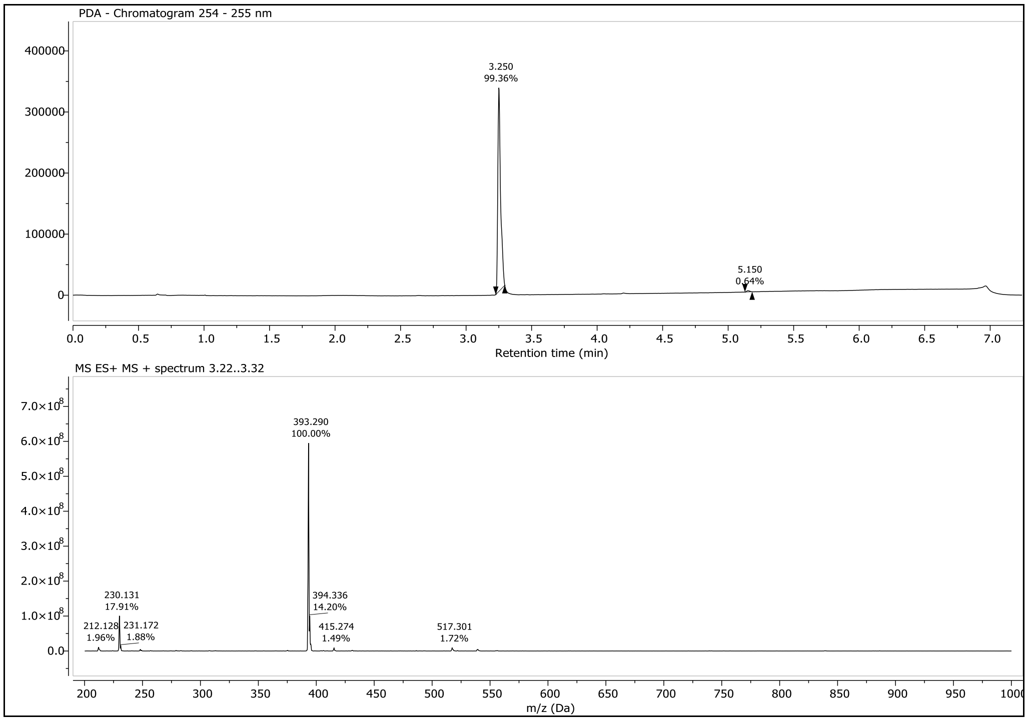
PDA - Chromatogram 254 - 255 nm
3.250
99.36%
5.150
0.64%
Retention time (min)
MS ES+ MS + spectrum 3.22..3.32
393.290
100.00%
230.131
17.91%
212.128
1.96%
231.172
1.88%
394.336
14.20%
415.274
1.49%
517.301
1.72%
m/z (Da)

(1-Methyl-2-oxo-1,2-dihydro-[3,3'-bipyridine]-5-carbonyl)-L-tyrosine (**3b**)

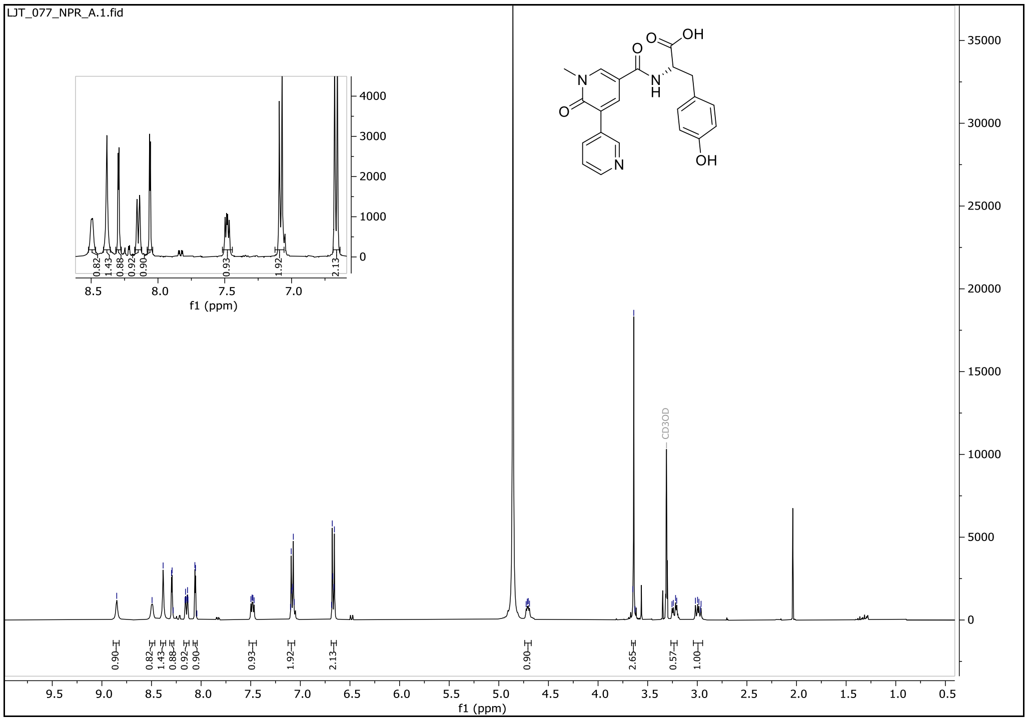

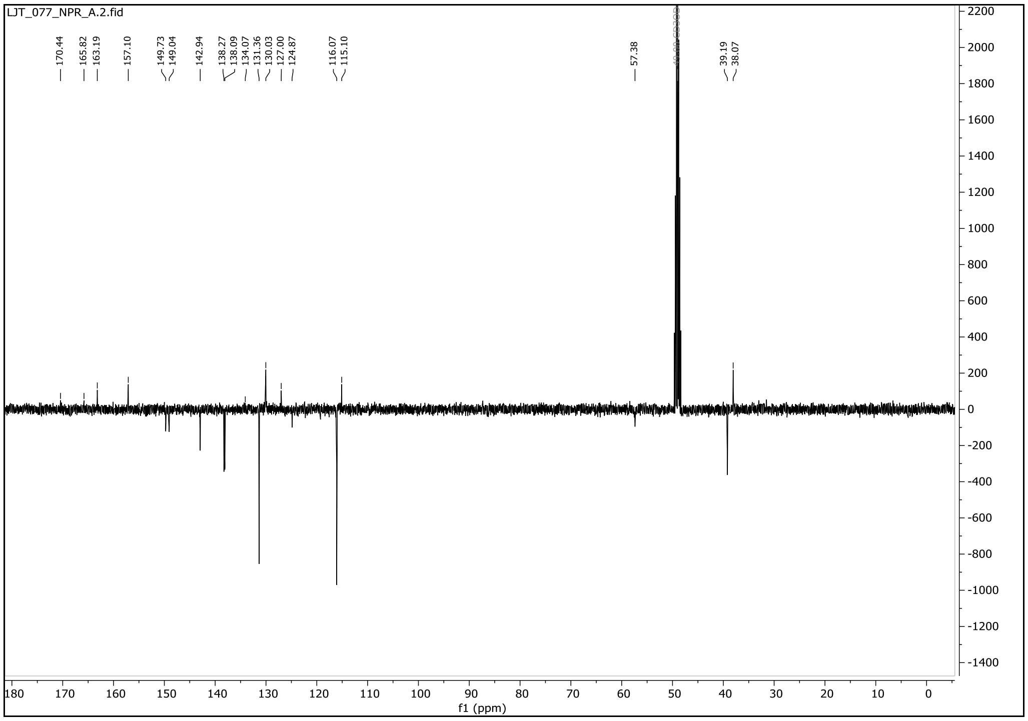

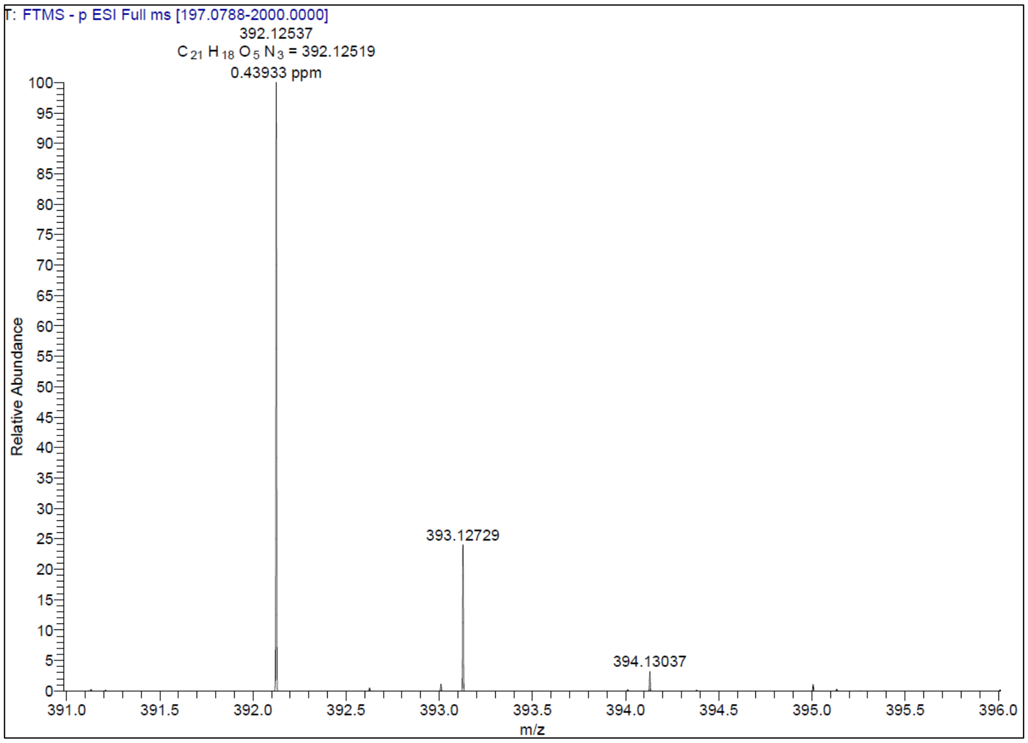
T: FTMS - p ESI Full ms [197.0788-2000.0000]
392.12537
C 21 H 18 O 5 N 3 = 392.12519
0.43933 ppm
393.12729
394.13037
Relative Abundance
m/z

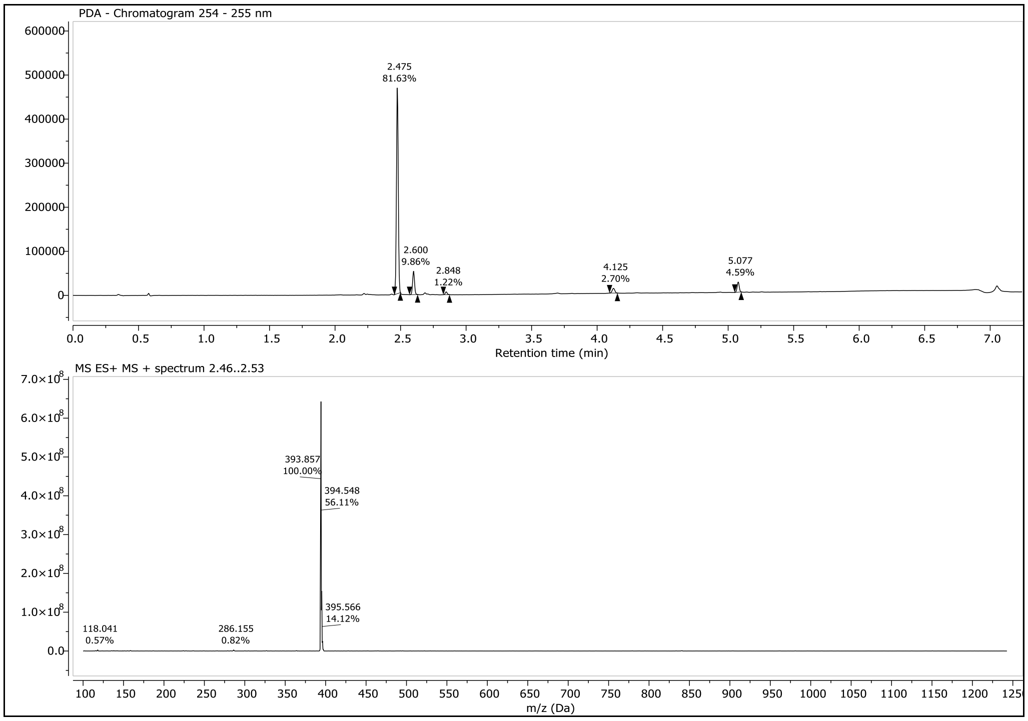
PDA - Chromatogram 254 - 255 nm
2.475
81.63%
2.600
9.86%
2.848
1.22%
4.125
2.70%
5.077
4.59%
Retention time (min)
MS ES+ MS + spectrum 2.46..2.53
393.857
100.00%
394.548
56.11%
395.566
14.12%
118.041
0.57%
286.155
0.82%
m/z (Da)

(1-Methyl-6-oxo-5-(thiophen-3-yl)-1,6-dihydropyridine-3-carbonyl)-L-tyrosine (**3c**)

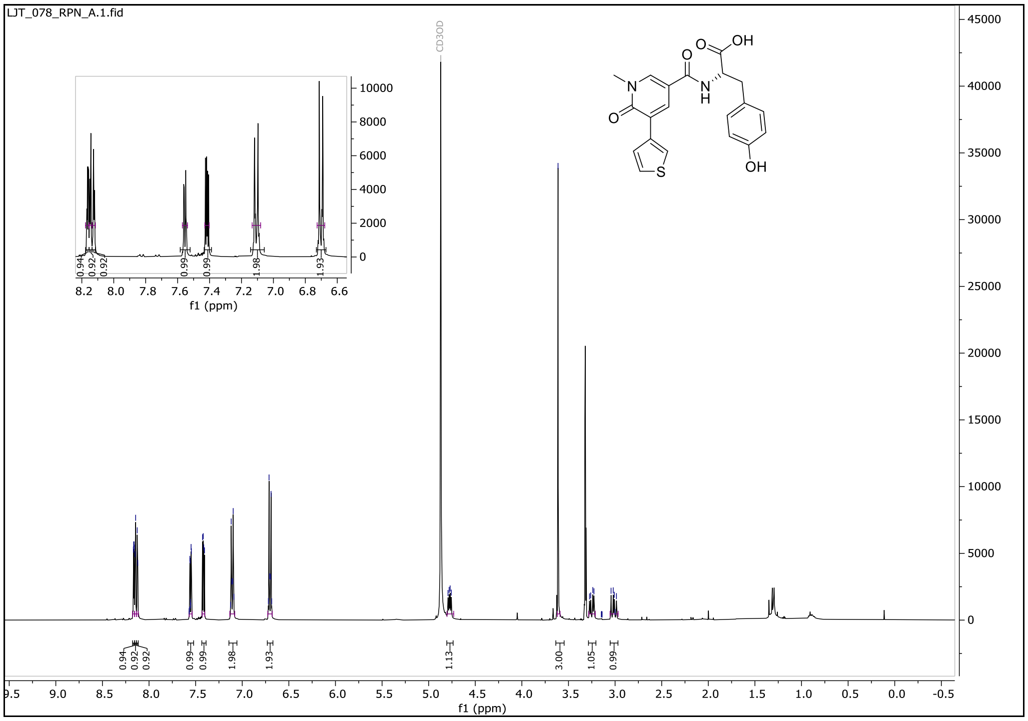

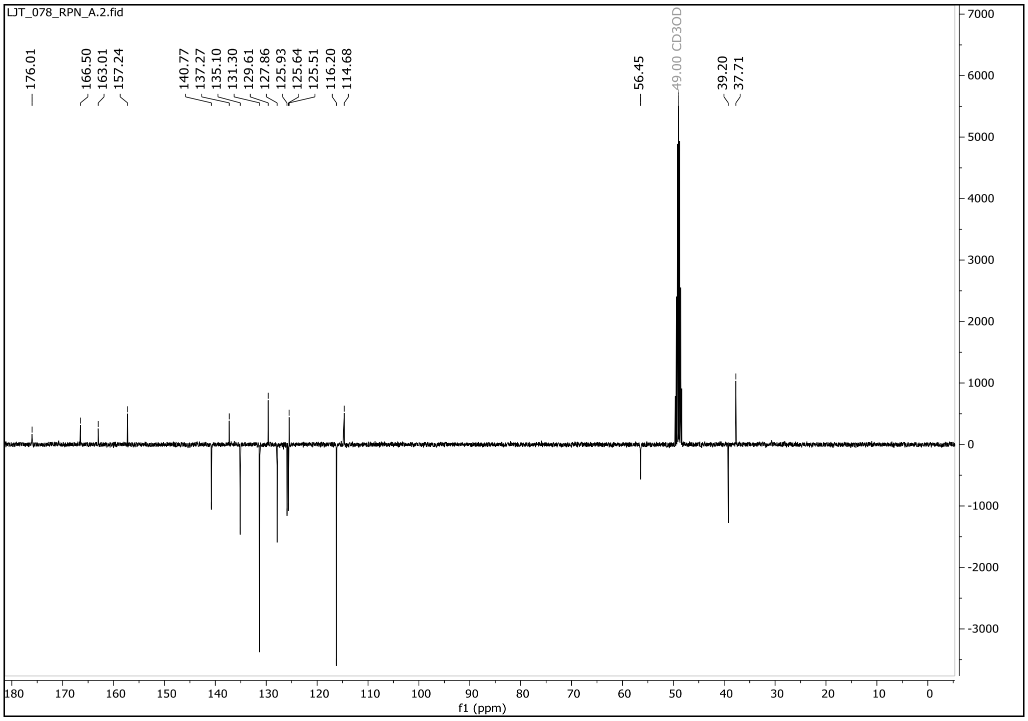

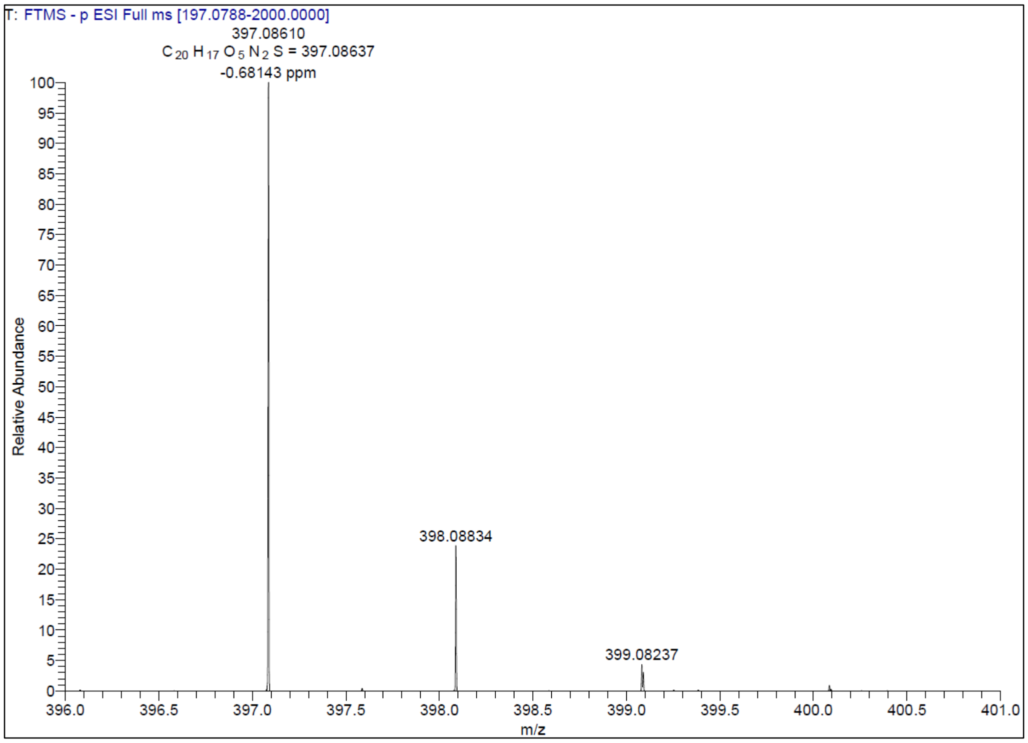
T: FTMS - p ESI Full ms [197.0788-2000.0000]
397.08610
$C_{20}H_{17}O_5N_2S$ = 397.08637
-0.68143 ppm
398.08834
399.08237
Relative Abundance
m/z

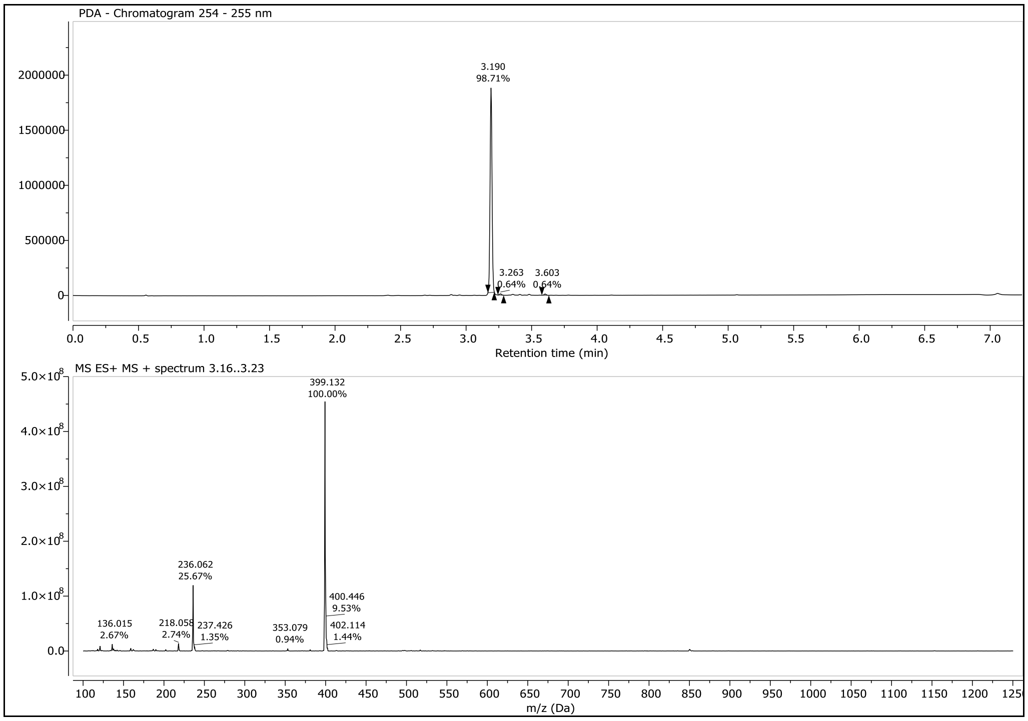
PDA - Chromatogram 254 - 255 nm
3.190
98.71%
3.263
0.64%
3.603
0.64%
Retention time (min)
MS ES+ MS + spectrum 3.16..3.23
399.132
100.00%
236.062
25.67%
400.446
9.53%
402.114
1.44%
136.015
2.67%
218.058
2.74%
237.426
1.35%
353.079
0.94%
m/z (Da)

(5-Oxo-6-phenyl-5H-thiazolo[3,2-a]pyridine-8-carbonyl)-L-tyrosine (**4a**)

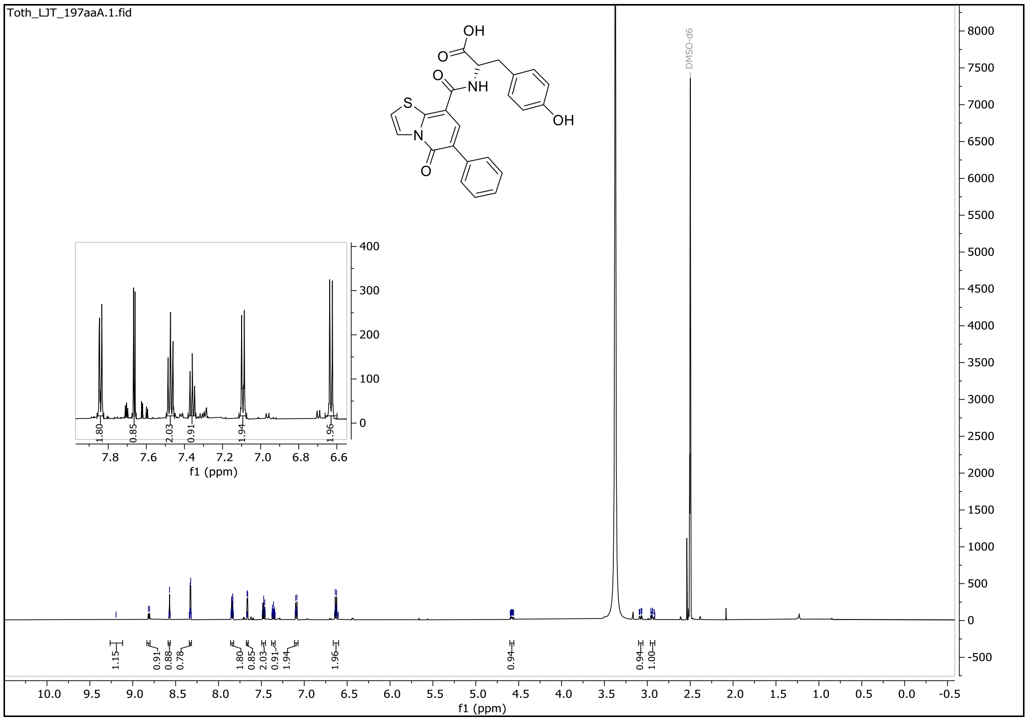

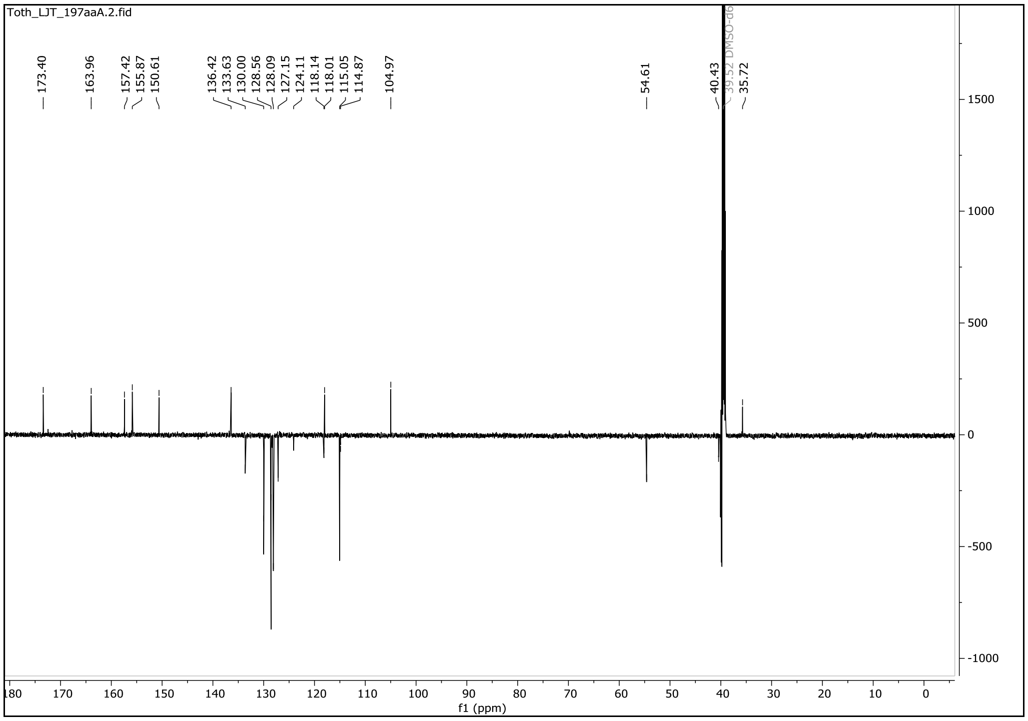

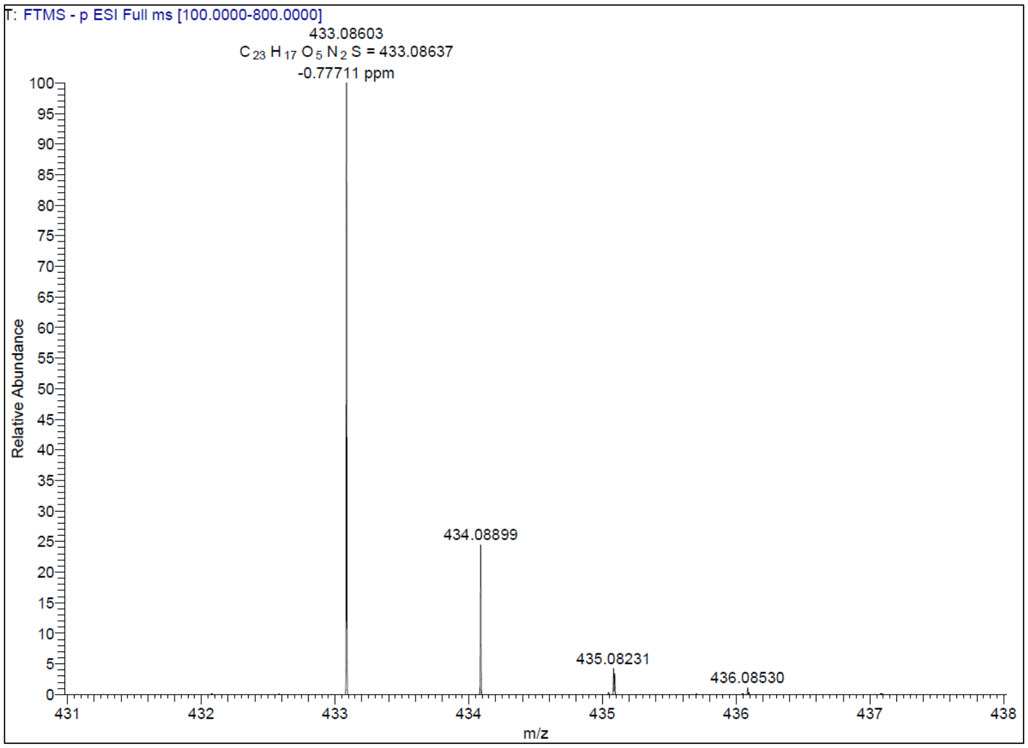
T: FTMS - p ESI Full ms [100.0000-800.0000]
433.08603
C 23 H 17 O 5 N 2 S = 433.08637
-0.77711 ppm
434.08899
435.08231
436.08530
Relative Abundance
m/z

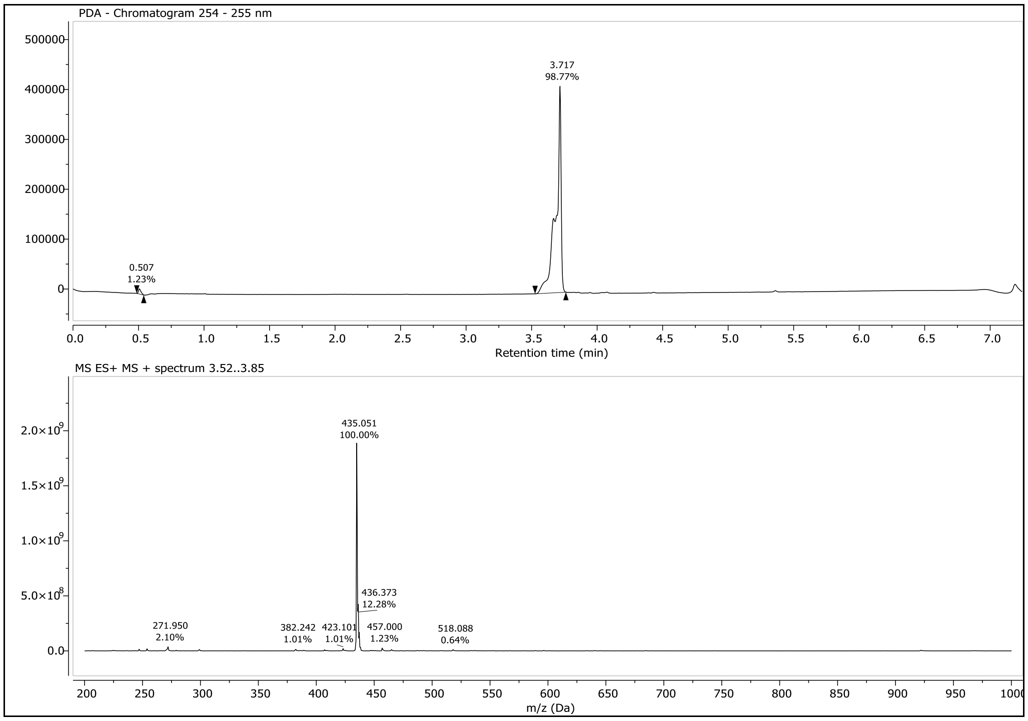
PDA - Chromatogram 254 - 255 nm
3.717
98.77%
0.507
1.23%
Retention time (min)
MS ES+ MS + spectrum 3.52..3.85
435.051
100.00%
436.373
12.28%
271.950
2.10%
382.242
1.01%
423.101
1.01%
457.000
1.23%
518.088
0.64%
m/z (Da)

(2-Methyl-5-oxo-6-phenyl-5*H*-[1,3,4]thiadiazolo[3,2-*a*]pyridine-8-carbonyl)-L-tyrosine (**4b**)

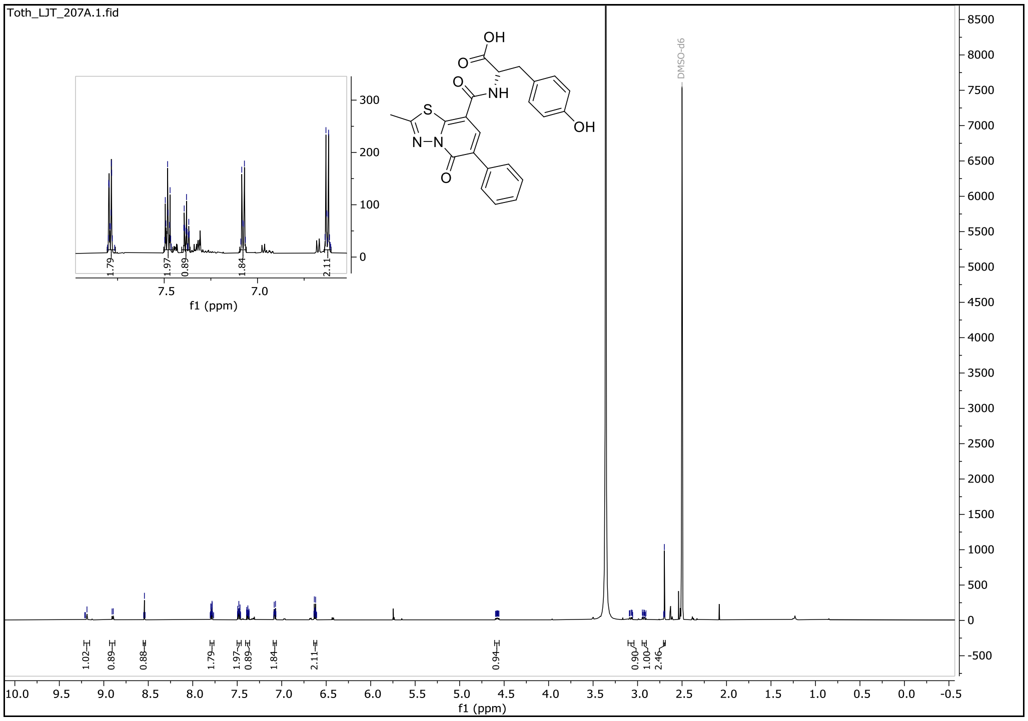

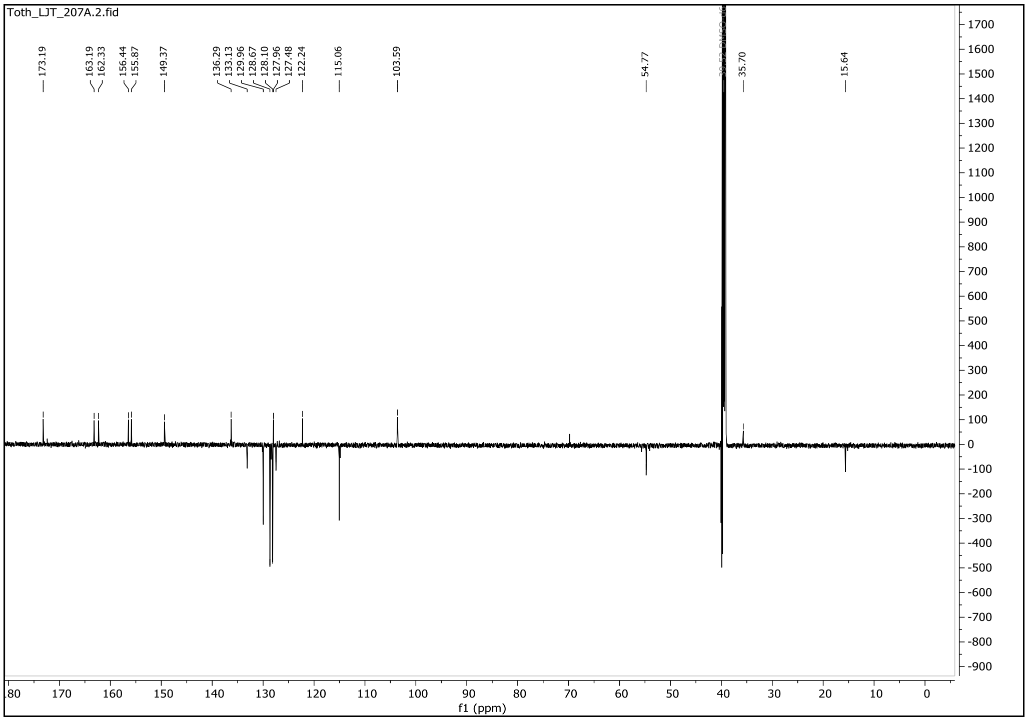

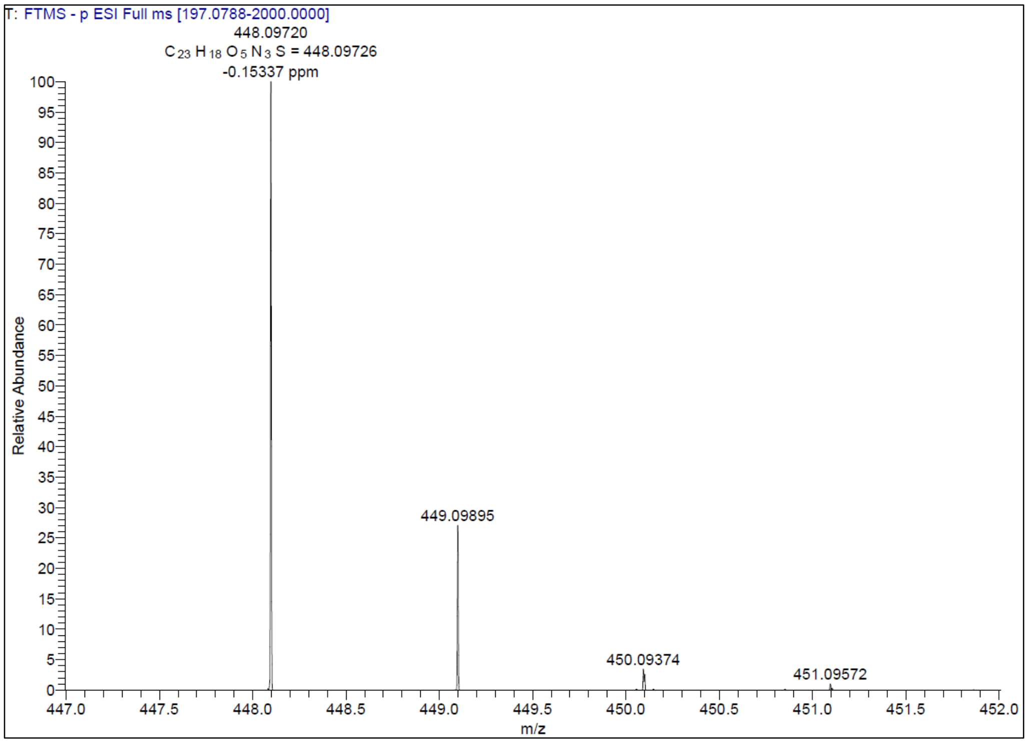
T: FTMS - p ESI Full ms [197.0788-2000.0000]
448.09720
C 23 H 18 O 5 N 3 S = 448.09726
-0.15337 ppm
449.09895
450.09374
451.09572
Relative Abundance
m/z

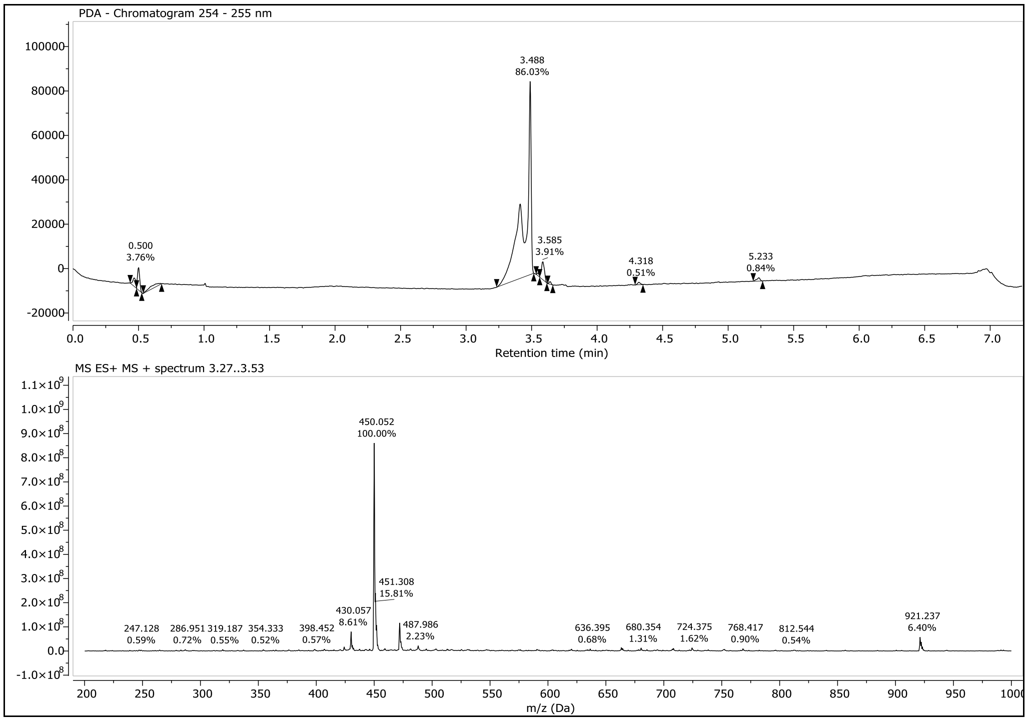
PDA - Chromatogram 254 - 255 nm
3.488
86.03%
0.500
3.76%
3.585
3.91%
4.318
0.51%
5.233
0.84%
Retention time (min)
MS ES+ MS + spectrum 3.27..3.53
450.052
100.00%
451.308
15.81%
430.057
8.61%
487.986
2.23%
247.128
0.59%
286.951
0.72%
319.187
0.55%
354.333
0.52%
398.452
0.57%
636.395
0.68%
680.354
1.31%
724.375
1.62%
768.417
0.90%
812.544
0.54%
921.237
6.40%
m/z (Da)

X-ray crystallography: crystal data and structure

The crystal data of **13** were collected using the Bruker D8 VENTURE system equipped with a Photon 100 CMOS detector, a multilayer monochromator and a MoKα Incoatec microfocus sealed tube (λ = 0.71073 Å) at 180 K. The frames were integrated with the with Bruker SAINT [8] software package. The structure was solved by direct methods with SIR92 [9] and were refined by full-matrix least-squares on F with CRYSTALS [10]. The positional and anisotropic thermal parameters of all nonhydrogen atoms were refined. All hydrogen atoms were located in a difference Fourier map, but those attached to carbon atoms were repositioned geometrically. The hydrogen atoms were initially refined with soft restraints on the bond lengths and angles to regularize the geometry, then their positions were refined with riding constraints.

Crystal data and crystal structure of compound **13** (colorless, 0.083 × 0.088 × 0.149 mm):

($C_{26}H_{27}N_1O_6$), triclinic, space group *P*-1, a°=°9.1751(4)°Å, b°=°10.7203(5)°Å, c°=°12.2177(6)°Å, α = 96.7702(18)°, *β* = 99.7218(16)°, γ = 103.8859(15)°, *V* = 1133.99(9) Å^3, *Z* = 2, *M* = 449.50, 120509 reflections measured, 5143 independent reflections. Final *R* = 0.045, *wR* = 0.043, *GoF* = 1.028 for 3897 reflections with $I > 2\sigma(I)$ and 299 parameters. CCDC 2309726.

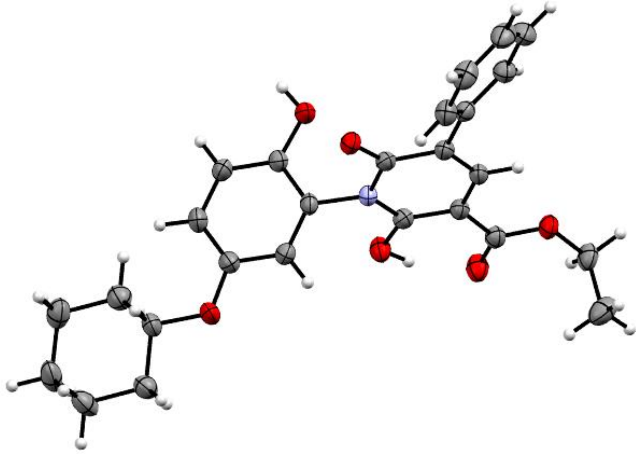

Figure S1: Crystal structure of compound **13**.

Biochemical study: inhibition of SARS-CoV-2 RdRp

Protein expression and purification

SARS-CoV-2 nsp12, nsp8 and nsp7 were expressed with appropriate purification and folding tags as described before [11–13]. Briefly, all of the proteins were expressed in bacterial cells, except for the full-length nsp12 protein, which was expressed in insect cells. The rest of the genes was expressed in *E. coli* (BL-21 CodonPlus (DE3)-RIL). Transformed cells were grown to an optimal OD_{600} in LB medium at 37 °C, the protein expression was induced by addition of IPTG to 0.4 mM, and the cells were grown at 18 °C for 20 h. The recombinant proteins were purified using Ni^{2+} affinity chromatography, followed by tag cleavage (when appropriate) and further purified using size exclusion chromatography.

Primer extension polymerase assay

The polymerase activity of the NS5 RdRp domain was determined in a primer extension reaction using a fluorescently labeled primer (HEX 5'- AGAACCUGUUGAACAAAAGC-3') and a template (5'-AUUAUUAGCUGCUUUUGU-3'). The reaction was performed in a reaction mixture containing 1 μM nsp12 polymerase, 3 μM nsp7, 3 μM nsp8, 0.5 μM template/primer complex and 10 μM NTPs in the reaction buffer (10 mM TRIS-HCl at pH 8.0, 10 mM KCl, 2mM $MgCl_2$, 1 mM β-mercaptoethanol) in a total volume of 10 µl. The reactions were incubated for 1 h at 30 °C with various concentrations of tested inhibitors and stopped by adding 20 µl of the stop buffer containing 80% formamide and 50 mM EDTA. The samples were denatured at 95 °C for 10 min, and primer extension products were separated on a 20% denaturing polyacrylamide gel (8 M urea, 1×TBE, 20% acrylamide (19:1)) and scanned on a Typhoon 5 Biomolecular Imager (GE Healthcare). The gels were analyzed using Imagequivuant TL 1D (version 8.2.0) program, and the curves and IC_{50} values were determined using the GraphPad algorithm.

Fluorescence-based primer extension assay of compound **2**

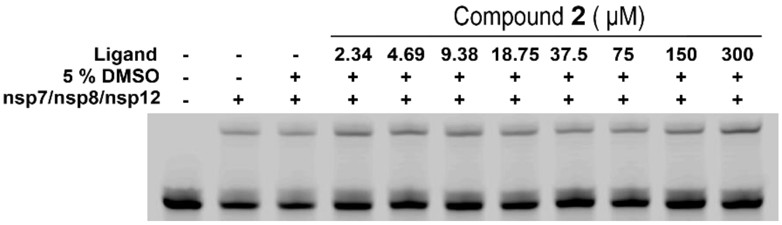

Figure S2: The result of the primer extension polymerase assay of compound **2**.

References

1. Nimnual, P.; Tummatorn, J.; Thongsornkleeb, C.; Ruchirawat, S., *J. Org. Chem.* **2015**, *80*, 8657-8667. doi: 10.1021/acs.joc.5b01305.

2. Jin, B.; Dong, Q.; Hung, G. Pyridinyl Based Apoptosis Signal-Regulating Kinase Inhibitors. WO 2018/151830 A1, 23.08.2018.

3. Serrano-Wu, M. H.; Hubbard, B. K.; Kaushik, V.; Daniels, D. PCSK9 Inhibitors and Methods of Use Thereof. WO 2020/150474 A1, 23.07.2020.

4. Winter-Holt, J. J.; Bardelle, C.; Chiarparin, E.; Dale, I. L.; Davey, P. R. J.; Davies, N. L.; Denz, C.; Fillery, S. M.; Guerot, C. M.; Han, F.; Hughes, S. J.; Kulkarni, M.; Liu, Z.; Milbradt, A.; Moss, T. A.; Niu, H.; Patel, J.; Rabow, A. A.; Schimpl, M.; Shi, J.; Sun, D.; Yang, D.; Guichard, S., *J. Med. Chem.* **2022**, *65*, 3306-3331. doi: 10.1021/acs.jmedchem.1c01871.

5. Potts, K. T.; Kanemasa, S. *J. Org. Chem.* **1979**, *44*, 3808-3811. doi: 10.1021/jo01336a014.

6. Walkinshaw, A. J.; Xu, W.; Suero, M. G.; Gaunt, M. J., *J. Am. Chem. Soc.* **2013**, *135*, 12532-12535. doi: 10.1021/ja405972h.

7. Dvorak, K. A.; Swanson, D. M.; Wong, V. D. Piperazinyl Derivatives Useful as Modulators of the Neuropeptide Y2 Receptor. U.S. Patent US8338426B2, June 26, 2008.

8. Inc., B. A. *SAINT*, Madison, Wisconsin, USA, 2015.

9. Altomare, A. C., G.; Giacovazzo G.; Guagliardi A.; Burla M. C.; Polidori, G.; Camalli, M., *J. Appl. Crystallogr.* **1994**, *27*, 435. doi: 10.1107/S002188989400021X.

10. Betteridge, P. W.; Carruthers, J. R.; Cooper, R. I.; Prout, K.; Watkin, D. J., *J. Appl. Crystallogr.* **2003**, *36*, 1487. doi: 10.1107/S0021889803021800.

11. Dejmek, M.; Konkolova, E.; Eyer, L.; Strakova, P.; Svoboda, P.; Sala, M.; Krejcova, K.; Ruzek, D.; Boura, E.; Nencka, R., *Viruses* **2021**, *13*, No. 1585. doi: 10.3390/v13081585.

12. Konkolova, E.; Krejcova, K.; Eyer, L.; Hodek, J.; Zgarbova, M.; Fortova, A.; Jirasek, M.; Teply, F.; Reyes-Gutierrez, P. E.; Ruzek, D.; Weber, J.; Boura, E., *Molecules* **2022**, *27*, No. 1894. doi: 10.3390/molecules27061894.

13. Konkolova, E.; Dejmek, M.; Hrebabecky, H.; Sala, M.; Boserle, J.; Nencka, R.; Boura, E., *Antiviral Res.* **2020**, *182*, No. 104899. doi: 10.1016/j.antiviral.2020.104899.
